# Antennal‐lobe neurons in the moth *Helicoverpa armigera*: Morphological features of projection neurons, local interneurons, and centrifugal neurons

**DOI:** 10.1002/cne.25034

**Published:** 2020-10-05

**Authors:** Jonas Hansen Kymre, Christoffer Nerland Berge, Xi Chu, Elena Ian, Bente G. Berg

**Affiliations:** ^1^ Chemosensory lab, Department of Psychology Norwegian University of Science and Technology Trondheim Norway; ^2^ Laboratory for Neural Computation, Institute of Basic Medical Sciences University of Oslo Oslo Norway

**Keywords:** AMIRA reconstruction, antennal‐lobe tracts, individual neurons, insect olfaction, intracellular staining, parallel pathways

## Abstract

The relatively large primary olfactory center of the insect brain, the antennal lobe (AL), contains several heterogeneous neuronal types. These include projection neurons (PNs), providing olfactory information to higher‐order neuropils via parallel pathways, and local interneurons (LNs), which provide lateral processing within the AL. In addition, various types of centrifugal neurons (CNs) offer top‐down modulation onto the other AL neurons. By performing iontophoretic intracellular staining, we collected a large number of AL neurons in the moth, *Helicoverpa armigera*, to examine the distinct morphological features of PNs, LNs, and CNs. We characterize 190 AL neurons. These were allocated to 25 distinct neuronal types or sub‐types, which were reconstructed and placed into a reference brain. In addition to six PN types comprising 15 sub‐types, three LN and seven CN types were identified. High‐resolution confocal images allowed us to analyze AL innervations of the various reported neurons, which demonstrated that all PNs innervating ventroposterior glomeruli contact a protocerebral neuropil rarely targeted by other PNs, that is the posteriorlateral protocerebrum. We also discuss the functional roles of the distinct CNs, which included several previously uncharacterized types, likely involved in computations spanning from multisensory processing to olfactory feedback signalization into the AL.

## Graphical abstract

The current study investigated morphological features of all three neuronal populations in the primary olfactory center of the moth, *Helicoverpa armigera*, by means of intracellular iontophoretic staining combined with confocal microscopy. The detailed characterizations of individual antennal‐lobe neurons—covering projection neurons, local interneurons, and centrifugal neurons—include several novel categories not previously described. Amira reconstructions were used to visualize the distinct neuronal types and sub‐types. Confocal images of all 190 neurons demonstrate the impressive morphological variety, suggesting complex signal processing already at this level of the insect olfactory pathway.

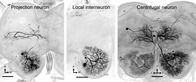

**Abbreviations**
AC: anterior cell body cluster
AL: antennal lobe
ALT: antennal‐lobe tract
AMMC: antennal mechanosensory and motor center
AOTU: anterior optic tubercle
AST: antenno‐subesophageal tract
bCa: anterior base of the calyces of the mushroom body
CN: centrifugal neuron
CRE: crepine
CSDn: contralaterally projecting serotonin‐immunoreactive deutocerebral neuron
DAAR: dopaminergic arching neuron
dALT: dorsal antennal‐lobe tract
dmALT: dorsomedial antennal‐lobe tract
GCBR: cell body rind lateral to the gnathal ganglion
ICL: inferior clamp
INP: inferior neuropils
LAL: lateral accessory lobe
lALT: lateral antennal‐lobe tract
LC: lateral cell body cluster
LH: lateral horn
LN: local interneuron
LPOG: labial palp‐pit organ glomerulus
mALT: medial antennal‐lobe tract
MC: medial cell body cluster
MCBR: cell body rind along the midline
MGC: macroglomerular complex
MIP: maximum intensity projection
mlALT: mediolateral antennal‐lobe tract
OGs: ordinary glomeruli
OSN: olfactory sensory neuron
PCx: posterior complex
PLP: posteriorlateral protocerebrum
PN: projection neuron
SCL: superior clamp
SEZ: subesophageal zone
SFS: superior fiber system
SIP: superior intermediate protocerebrum
SLP: superior lateral protocerebrum
SMP: superior medial protocerebrum
tALT: transverse antennal‐lobe tract
TKir: tachykinin‐immunoreactive
VLP: ventrolateral protocerebrum
VMNP: ventromedial neuropils
VPGs: ventroposterior glomeruli

## 1 INTRODUCTION

Insects use chemical signals for a wide variety of functions including foraging, finding a mate, locating oviposition habitats, and avoiding predators (reviewed by Berg, Zhao, & Wang, 2014; Reisenman, Riffell, & Hildebrand, 2009). The neuroanatomical structures underlying these odor‐driven behaviors are remarkably similar across phylogenetically distant insect species (reviewed by Galizia & Rössler, 2010). Generally, the primary olfactory brain center, the antennal lobe (AL), receives direct input about the chemical environment via numerous olfactory sensory neurons (OSNs) located on the antennae. In the AL, OSNs expressing the same odor‐ligand receptor converge onto distinct spherical neuropil structures called glomeruli, creating a chemosensory map (Vosshall, Wong, & Axel, 2000).

The AL of the noctuid moth, *Helicoverpa armigera* (Lepidoptera: *Noctuidae*, *Heliothinae*), comprises 79 glomeruli forming distinct AL sub‐systems (Zhao et al., 2016)—the relatively numerous putatively general‐odor sensitive ordinary glomeruli (OGs), the male‐specific macro‐glomerular complex (MGC), the yet functionally undescribed posterior complex (PCx), and the CO_2_‐responsive labial palp‐pit organ glomerulus (LPOG). In addition, a group of ventroposteriorly located AL glomeruli have shown distinct forms of sensory detection in many other insects. In the fruit fly, *Drosophila melanogaster*, the cockroach, *Periplaneta americana*, and the honeybee, *Apis mellifera*, several ventroposterior glomeruli (VPGs) are involved in hygro‐ and thermo‐sensory processing (Enjin et al., 2016; Frank et al., 2017; Frank, Jouandet, Kearney, Macpherson, & Gallio, 2015; Nishino et al., 2003; Nishino, Nishikawa, Mizunami, & Yokohari, 2009). Currently, corresponding glomeruli have not been functionally characterized in moths, and were until recently not considered to belong to the AL (Skiri, Rø, Berg, & Mustaparta, 2005; Zhao et al., 2016).

As in other insects, three categories of AL neurons partake in information processing: (a) projection neurons (PNs) conveying odor information from the AL to higher brain centers, (b) local interneurons (LNs) important for AL‐internal processing, and (c) centrifugal neurons (CNs) conveying top‐down information into the AL (reviewed by Homberg, Christensen, & Hildebrand, 1989). Somata of PNs and LNs form three peripherally located cell body clusters encapsulating the glomeruli, that is the lateral, medial, and anterior cell body clusters (LC, MC, and AC, respectively). All LNs have their somata in the large LC, while somata of PNs are located in either the LC, MC, or AC (Homberg, Montague, & Hildebrand, 1988). Cell bodies of CNs are usually positioned outside the AL.

**FIGURE 1:**
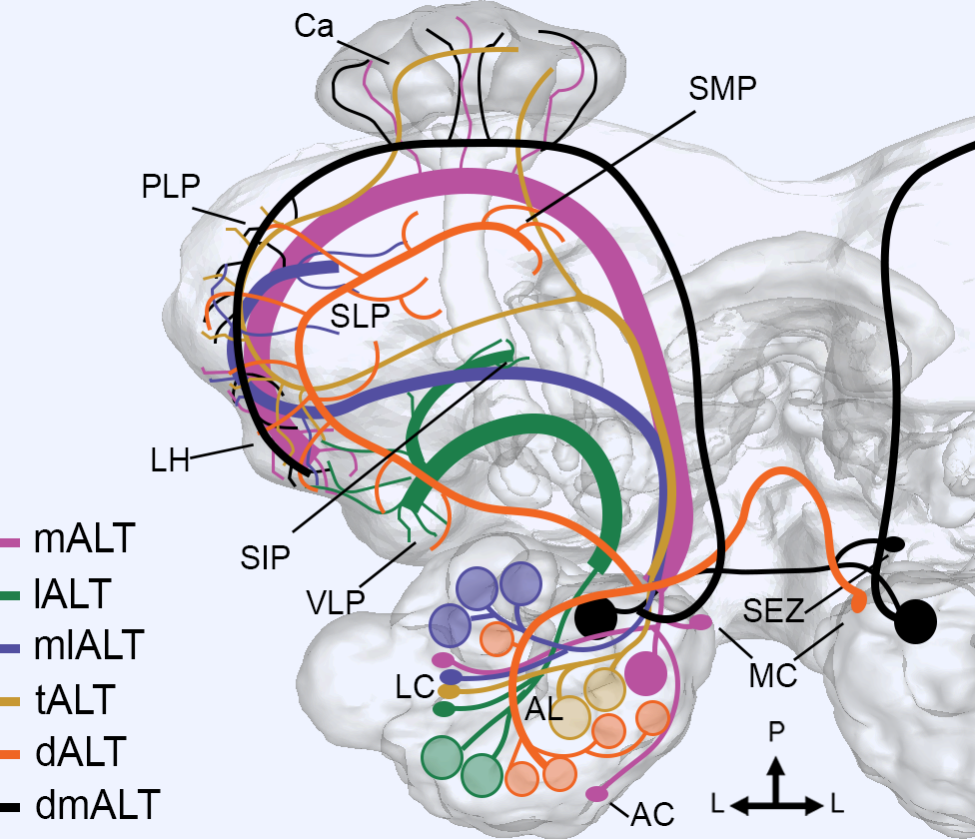
Schematic view of the parallel antennal‐lobe tracts (ALTs) formed by projection neurons (PNs). The PNs of the antennal lobe (AL), following one of six distinct ALTs, vary in axonal projection patterns, glomerular arborizations, and soma location. Here, the thickness of each ALT roughly relates to its estimated number of PNs. The medial‐tract (mALT) PNs target the calyces (Ca) and lateral horn (LH), and have somata in all three AL cell clusters, that is the anterior, lateral, and medial clusters (AC, LC, and MC, respectively). The lateral‐tract (lALT) PNs have more heterogeneous projection patterns, including output areas in the ventrolateral protocerebrum (VLP), LH, and the column of the superior intermediate protocerebrum (SIP). The mediolateral‐tract (mlALT) PNs typically innervate the LH and the superior lateral protocerebrum (SLP), but may also target other neuropils, for example, the posteriorlateral protocerebrum (PLP). Transverse‐tract (tALT) PNs have varying target regions, including the Ca, LH, and PLP. PNs of the lALT, mlALT and tALT all have somata in the LC. The dorsal‐tract (dALT) PNs have their somata in the contralateral MC, and target many protocerebral neuropils, including the VLP, LH, PLP, SLP, and the superior medial protocerebrum (SMP). Bilateral dorsomedial (dmALT) PNs have somata in the subesophageal zone (SEZ), innervate one glomerulus in the AL of each hemisphere, and project bilaterally to the Ca, PLP and LH. Glomerular arborization pattern differs between ALTs; the PNs may be uniglomerular (mostly mALT and dmALT), oligoglomerular (tALT, lALT, and mlALT), or arborizing in most glomeruli (dALT and some PNs from the lALT, and mlALT). L, lateral; P, posterior [Color figure can be viewed at wileyonlinelibrary.com]

The PNs constitute a heterogeneous neuronal group carrying odor information from the AL to higher brain centers via several parallel antennal‐lobe tracts (ALTs). Altogether, moths have ~850 PNs restricted to six ALTs in each brain hemisphere, that is the medial, mediolateral, lateral, transverse, dorsomedial, and dorsal ALT (m‐, ml‐ l‐, t‐ dm‐, and d‐ALT, respectively; see Figure 1). As the majority of the PNs follow the first three ALTs, they are considered the main tracts (Homberg et al., 1988; Ian, Berg, Lillevoll, & Berg, 2016). PNs principally target two main areas—the calyces of the mushroom bodies and the lateral protocerebrum, including the lateral horn (LH; Homberg et al., 1988; Ian, Zhao, Lande, & Berg, 2016). So far, uniglomerular PNs in the mALT, which innervate the two former protocerebral areas, are most frequently reported. The PNs following the mALT have their somata located in one of the three cell body clusters, whereas PNs confined to the other main tracts have their somata in the LC (Homberg et al., 1988). The dendritic and axonal arborization patterns of individual PNs within any given ALT are quite diverse (see Table 1 for summary of previous reports in moths). Actually, this diversity of PN types is rather common in insects. Even the small fruit fly, having only ~350 PNs (Bates et al., 2020), possesses at least 15 different PN sub‐types (Tanaka, Endo, & Ito, 2012).

**TABLE 1:** Previously reported PN morphologies in Lepidoptera

ALT	Sub‐type	AL innervation	Non‐AL innervation	Soma	References
**mALT**	Pm_a	**UG**; MG	Ca; LH; SLP^a^	A‐, L‐, MC	1, 3–7, 9–13, 15–16
	Pm_b ^b^	‐	SLP; LH	LC	4
	Pm_c ^b^	MG	LH; Ca	LC	4, 12
	Pm_d	UG	Ca; LH; VLP; INP	LC	5
	Pm_e	MG	Ca; LH; INP; SNP; VMNP; VLP	AC	5
	Pm_f	MG	CL. VMNP; PLP	LC	5
**lALT**	Pl_a_uni	MG	AL Isthmus; Col.	LC	2–5, 8
	Pl_a_bi	UG; MG	Col.; LH; PED; CL. Col.	LC	2, 5, 8, 14
	Pl_b	MG	VLP	LC	4, 12
	Pl_c	UG; MG	LH; Ca	LC	3–4, 10, 12
	Pl_d	UG; MG	AMMC; SEZ; VLP; TC; LH	LC	4
	Pl_e	MG	VLP; SLP	LC	5
	Pl_f	MG	LH; INP	LC	5
**mlALT**	Pml_a	MG	VLP; LH	LC	4–5, 8, 12
	Pml_b	UG; **MG**	LH; SLP	LC	4–5, 10, 12
**tALT**	Pt	MG	LH; INP; VLP; SEZ	LC	5
**dmALT**	Pdm	UG UL. & BL.	Ca; LH UL. & BL.	GCBR	5–6, 10, 12
**dALT**	Pd	MG	LH	CL. MC	4, 6, 10

*Note*: Innervations in bold are most frequent. References: 1) Anton, Löfstedt, and Hansson (1997); 2) Chu, Heinze, Ian, & Berg, (2020); 3) Hansson, Anton, and Christensen (1994); 4) Homberg et al. (1988); 5) Ian, Zhao, et al. (2016); 6) Kanzaki, Arbas, Strausfeld, and Hildebrand (1989); 7) Kanzaki, Soo, Seki, and Wada (2003); 8) Lee, Celestino, Stagg, Kleineidam, and Vickers (2019); 9) Løfaldli, Kvello, and Mustaparta (2010); 10) Namiki and Kanzaki (2011); 11) Nirazawa et al. (2017); 12) Rø, Müller, and Mustaparta (2007); 13) Sadek, Hansson, Rospars, and Anton (2002); 14) Wu, Anton, Löfstedt, and Hansson (1996); 15) Zhao and Berg (2010); 16) Zhao et al. (2014).

^a^ indicates the region formerly known as the inferior lateral protocerebrum (i.e., limited to PNs innervating the macroglomerular complex).

^b^ These neurons project through the recently identified transverse tract.

Abbreviations: AC, anterior cell cluster; AL, antennal lobe; ALT, antennal‐lobe tract; AMMC, antennal mechanosensory and motor center; BL., bilateral; Ca, calyces; CL., contralateral; Col., column of the superior intermediate protocerebrum; dALT, dorsal ALT; dmALT, dorsomedial ALT; GCBR, cell body rind around the gnathal ganglion; INP, inferior neuropil; lALT, lateral ALT; LC, lateral cell cluster; LH, lateral horn; mALT, medial ALT; MC, medial cell cluster; MG, multiglomerular; mlALT, mediolateral ALT; PED, peduncle of the mushroom body; PLP, posteriorlateral protocerebrum; SEZ, subesophageal zone; SLP, superior lateral protocerebrum; SNP, superior neuropil; tALT, transverse ALT; TC, tritocerebrum; UL., unilateral; UG, uniglomerular; VLP, ventrolateral protocerebrum; VMNP, ventromedial neuropil.

The LNs are axon‐less, spatially confined neurons having both input and output synapses in the AL (Christensen, Waldrop, Harrow, & Hildebrand, 1993; Tabuchi et al., 2015). Most LNs are GABAergic (Berg, Schachtner, & Homberg, 2009; Reisenman, Dacks, & Hildebrand, 2011; Seki & Kanzaki, 2008), providing inhibition to the AL network. Many LNs innervate most of the glomeruli, and were termed MGC‐AllGs LNs by Seki and Kanzaki (2008). Oligoglomerular LNs innervating a subset of the glomeruli have also been reported (Christensen et al., 1993; Reisenman et al., 2011; Sadek et al., 2002; Seki & Kanzaki, 2008).

In comparison with PNs and LNs, the CNs pose a minor category. These neurons extend dendrites outside the AL, and have projection terminals within the AL. Generally, the CNs modulate signal transmission in the afferent pathway (reviewed by Schachtner, Schmidt, & Homberg, 2005). One of the most thoroughly studied CNs, the *contra‐laterally projecting serotonin‐immunoreactive deutocerebral neuron* (CSDn), first described in the sphinx moth, *Manduca sexta* (Kent, Hoskins, & Hildebrand, 1987), can enhance PN responses to biologically relevant odor stimuli (Kloppenburg, Ferns, & Mercer, 1999). Only a few CN types other than the CSDn have been reported in Lepidoptera so far; a unilateral multisensory CN (Zhao, Pfuhl, Surlykke, Tro, & Berg, 2013), a bilateral dopaminergic arching (DAAR) neuron (Dacks, Riffell, Martin, Gage, & Nighorn, 2012; Lizbinski, Metheny, Bradley, Kesari, & Dacks, 2016), and a tachykinin‐immunoreactive CN connecting the AL and the antennal mechanosensory and motor center (AMMC; Berg, Schachtner, Utz, & Homberg, 2007).

To extend our understanding of how odor signals are processed in the AL and how this information is conveyed to higher brain centers, we have elucidated the structure of the basic coding element in this system, that is the individual *neuron*. By using iontophoretic staining, combined with confocal microscopy, we have systematically gathered anatomical data from individual neurons in the heliothine moth, *H. armigera*. The total collection of 190 AL neurons identified and presented here, covers all three categories—PNs, LNs, and CNs. In addition to previously reported neuron types, we present several novel ones. Altogether, the data presented here provide a comprehensive and detailed overview of the neurons forming the primary olfactory processing center.

## 2 MATERIALS AND METHODS

### 2.1 Insect preparation

Pupae of *H. armigera* were obtained from Keyun Bio‐pesticides (Henan, China). All neurons were sampled from males, unless otherwise noted. The insects were kept in climate chambers at 23°C and 70% humidity, with reversed day‐night cycle (light–dark 14:10 hr). After emergence, the moths were provided a 10% sucrose solution. According to Norwegian law of animal welfare there are no restrictions regarding experimental use of Lepidoptera.

The moth was placed in a plastic tube, where the protruding head was immobilized using a layer of dental wax (Kerr Corporation, Romulus, MI). To access the brain, a part of cuticle and antennal muscles were carefully removed. Ringer solution (in mM: 150 NaCl, 3 CaCl2, 2 KCl, 25 sucrose, and 10 N‐tris [hydroxymethyl]‐methyl‐2‐amino‐ethanesulfonic acid, pH 6.9) was applied continuously during the experiments.

### 2.2 Intracellular staining and confocal microscopy

The staining procedure was performed as previously described (Chu et al., 2020; Ian, Zhao, et al., 2016; KC et al., 2020). Electrodes were pulled from borosilicate capillaries (OD: 1 mm, ID: 0.5 mm, with hollow filament 0.13 mm; Hilgenberg GmbH, Germany) using a horizontal P‐97 Flaming/Brown Pipette Puller (Sutter Instruments Inc.). Electrode‐tips were back‐filled with 4% biotinylated dextran‐conjugated tetramethylrhodamine (3,000 mw, micro‐ruby, Molecular Probes; Invitrogen, Eugene, OR) in 0.2 M KAc or 4% Alexa Fluor 488 (10,000 mw, Molecular Probes; Invitrogen, Eugene, OR) in distilled water, and further filled with 0.2 M KAc. The dye‐filled electrode was appended to a silver wire attached to a head‐stage (HS‐2, Axon instruments), which was connected to an amplifier (Axoprobe‐1A, Axon instruments). By means of a Leica micromanipulator, the electrode was maneuvered into AL neurons. Staining electrode resistance in the Ringer solution was generally 70–200 MΩ. A chlorinated silver reference electrode was inserted into the moth's mouth muscles. Iontophoretic staining of a contacted cell was performed by injecting 1.5–3.0 nA depolarizing current pulses of 200 ms duration at 1 Hz for 5–15 minutes. After staining, the preparation was kept overnight at 4°C to facilitate dye‐transportation. The brain was then dissected out and stored in 4% paraformaldehyde (Roti‐histofix 4%, Carl Roth GmbH, Karlsruhe, Germany) for 1 h at room temperature. Afterwards, the brain was dehydrated in an increasing ethanol series (50%, 70%, 90%, 96%, 2 x 100% for 10 minutes each). Finally, the preparation was mounted in 1 mm aluminium plates with methyl salicylate (methyl 2‐hydroxybenzonate; Merck KGaA, Germany) and sealed by coverslips.

The brains were scanned by means of confocal microscopy (Zeiss LSM 800, Jena, Germany) using either of two objectives: a 10x/0.45 water objective (C‐achroplan) or a 20x/0.5 air (Pan Neofluar). A 553 nm HeNe laser excited neurons labeled with micro‐ruby, while emission was collected with a 568 long‐pass filter. Neurons filled with Alexa Fluor 488 were excited by a 493 nm Argon laser, and emission was captured by a 505–550 nm band pass filter. Optical slice distance was between 2–8 μm. Images were processed in Zen 2.3 blue edition (Carl Zeiss Microscopy, GmbH, Jena, Germany) and MATLAB R2018b.

### 2.3 Reconstruction

Digital reconstruction of individual neurons, acquired by confocal scanning, was used to visualize individual neurons. In some cases, reconstruction also aided visualization of weakly stained filaments. The individual neuron was traced manually by using the SkeletonTree plugin (Evers, Schmitt, Sibila, & Duch, 2005; Schmitt, Evers, Duch, Scholz, & Obermayer, 2004) in 3D reconstruction software Amira 5.3 (Visualization Science Group). To compensate for refraction indexes, the z‐axis dimension of the brain was multiplied by a factor of 1.16 for the water objective and 1.54 for the air lens. Reconstructed neurons were manually transformed into the 3D representative brain of *H. armigera* (Chu et al., 2020) in accordance with their original projection pattern.

### 2.4 Nomenclature and neuronal classification

The nomenclature was based on the work of the Insect Brain Name Working Group (Ito et al., 2014), which used the fruit fly as a model organism. Determining the position of distinct neuropils was based upon inspection of 3D models of standardized brains from the fruit fly (Ito et al., 2014) and the monarch butterfly, *Danaus plexippus* (Heinze, 2016), but adjusted in accordance with landmarks in the moth brain. The classification of PNs also built on previous naming systems used in the moth species, *M*. *sexta* (Homberg et al., 1988) and *Heliothis virescens* (Ian, Zhao, et al., 2016). We excluded PNs classified exclusively as MGC‐ or LPOG‐neurons, these will be presented in separate studies. In addition, electrophysiological data will be presented in an upcoming article.

For multiglomerular neurons, classification of dendritic morphology was based on the nomenclature for LNs in the silkmoth, *Bombyx mori* (Seki & Kanzaki, 2008). Identification of specific AL glomeruli and nerve bundles followed the arrangement specified for *H. armigera* (Zhao et al., 2016), except for the ventroposteriorly located glomeruli, the VPGs. Our new definition of a glomerular group called the VPGs is based on the finding of an assembly of heteromorphic glomerular units that were frequently interconnected by oligoglomerular PNs arborizing primarily or exclusively in these specific glomeruli. Furthermore, these glomeruli were weakly labeled by previous OSN mass dye fills (Zhao et al., 2016). The VPGs are proposed to include at least three glomeruli located posteriorly to the LPOG, that is G71‐G73, as well as G64‐G69, which form a horseshoe‐shaped cluster of undersized glomeruli ventral to the AL hub.

In some cases, distinguishing between CNs and PNs was complicated due to combinations of smooth and varicose processes in the same brain regions. Such structures are associated with post‐ and presynaptic sites, respectively (Cardona et al., 2010). In these instances, soma location was taken into consideration, in the sense that a soma found in the protocerebrum would indicate that the neuron was centrifugal.

## 3 RESULTS

We present a collection of individually labeled neurons consisting of all three AL neuron categories, that is PNs, LNs, and CNs. Morphological features are reported for a total of 109 successfully stained PNs, 67 LNs, and 14 CNs. Information on the anatomy of these three AL neuron categories is shown in Table 2, 3, 4, while innervations of individual PNs and LNs are described in Supplemental Table S1 & S2, respectively. All neurons are presented as maximum intensity projection confocal images and typical examples of all neuronal types are available as AMIRA reconstructions. In addition, selected neurons can be accessed via the Insect brain database (insectbrainDB, 2020).

**TABLE 2:** Overview of labeled projection neurons

ALT	Sub‐type	AL innervation	Non‐AL innervation	Soma	*N*
**mALT**					67
	Pm_a				61
		UG	Ca; LH	AC, LC, MC	55
		BG	Ca; LH	LC, MC	4
		OligoGs	Ca; LH	AC	2
	Pm_e	OligoGs	SEZ; AMMC; SLP; ICL; PLP; LH; VLP	AC	6
**lALT**					18
	Pl_b				2
		UG	VLP	LC	1
		OligoGs	VLP; VMNP	LC	1
	Pl_c				6
		UG	LH; PLP; Ca	LC	1
		OligoGs	AL isthmus; LH; PLP; Ca	LC	5
	Pl_d				6
		OligoGs	SEZ; AMMC; VMNP; VLP	LC	3
		MGC‐AllGs	SEZ; LAL; VMNP; VLP	LC	3
	Pl_e				4
		UG	VLP; SCL	LC	2
		OligoGs	SEZ; AMMC; VLP; LH; ICL	LC	2
**mlALT**					13
	Pml_b	OligoGs	LH; SLP; SMP; bCa	LC	4
	Pml_c				5
		UG	LH, PLP; SLP, ICL, bCa	LC	1
		OligoGs	LH, PLP; SLP, ICL, bCa	LC	4
	Pml_d	MGC‐AllGs	VLP; LH; SLP; SIP; SMP; CRE	LC	4
**tALT**					6
	Pt_a	OligoGs	LH; PLP; Ca	LC	3
	Pt_b	UG	SEZ; AMMC; BL. Ca; PLP; SLP	LC	1
	Pt_c	OligoGs	SLP; PLP	LC	1
	Pt_d	OligoGs	SLP; PLP	LC	1
**dmALT**					4
	Pdm	UG	BL. Ca; LH; PLP	GCBR	4
**dALT**					1
	Pd	MGC‐AllGs	CL. VLP; PLP; LH; bCa; SLP; SIP; SMP	CL. MC	1

Abbreviations: AC, anterior cell cluster; AL, antennal lobe; ALT, antennal‐lobe tract; AMMC, antennal mechanosensory and motor center; bCa, base of the calyces; BG, bi‐glomerular; BL., bilateral; Ca, calyces; CL., contralateral; CRE, crepine; dALT, dorsal ALT; dmALT, dorsomedial ALT; GCBR, cell body rind around gnathal ganglion; ICL, inferior clamp; lALT, lateral ALT; LC, lateral cell cluster; LH, lateral horn; mALT, medial ALT; MC, medial cell cluster; MGC‐AllGs, macroglomerular complex and all/most other glomeruli; mlALT, mediolateral ALT; OligoGs, oligoglomerular; PLP, posteriorlateral protocerebrum; PN, projection neuron; SCL, superior clamp; SEZ, subesophageal zone; SIP, superior intermediate protocerebrum; SLP, superior lateral protocerebrum; SMP, superior medial protocerebrum; tALT, transverse ALT; UG, uniglomerular; VLP, ventrolateral protocerebrum; VMNP, ventromedial neuropil.

**TABLE 3:** Overview of labeled antennal‐lobe local interneurons

Type	Glomerular innervation	PCx	MGC	LPOG	VPGs	OGs
MGC‐AllGs (*N* = 60)						
	Normal	47	41	42	46	60
	Sparse	11	18	10	8	–
	Dense	1	–	–	3	–
	Not innervated	1	–	5	–	–
	Gs not visible	–	1	3	3	–
OligoGs (*N* = 3)						
	Normal	–	–	1	1	3
	Sparse	1	1	–	1	–
	Not innervated	2	2	2	1	–
MGC‐AllGs‐IST (*N* = 4)						
	Normal	4	4	2	2	4
	Sparse	–	–	2	2	–

*Note*: Normal innervation within a sub‐system is here defined as most or all glomeruli within the specific sub‐system receiving processes that are comparable to that of the ordinary glomeruli (OGs). AL, antennal lobe; Gs, glomeruli; IST, antennal lobe isthmus; LN, local interneuron; LPOG, labial‐palp pit organ glomerulus; MGC‐AllGs, macroglomerular complex and all/most other glomeruli; OligoGs, oligoglomerular; PCx, posterior complex; VPGs, ventroposterior glomeruli.

**TABLE 4:** Overview of individual centrifugal neurons

Type (ID)	*N*	Soma	AL innervations	Non‐AL innervations	Figure
CSD (CN1)	1	LC	CL. MGC‐AllGs	BL. SMP; SLP; CBU; PLP; LH	10b
CSD (CN2)	1	LC	CL. MGC‐AllGs	BL. SMP; SLP; CBU; PLP; LH	12
DAAR (CN3)	1	PCBR	MGC‐AllGs	BL. SMP; CRE; SIP; SLP; AOTU	12
DAAR (CN4)	1	PCBR	MGC‐AllGs	BL. SMP; CRE; SIP; SLP; AOTU	10a
DAAR (CN5)	1	PCBR	MG^a^	BL. SMP; CRE; SIP^a^	12
Cm (CN6)	1	PCBR	MGC‐AllGs	LH; VLP; PLP; SLP; SIP; SMP; Ca; ML; CRE	12
Cm (CN7)	1	PCBR	MGC‐AllGs htg.	SCL; LH; VLP; PLP; SLP; SIP; SMP; Ca; ML; CRE	11a
Cm (CN8)	1	PCBR	MGC‐AllGs htg.	SCL; LH; VLP; PLP; SLP; SIP; SMP; Ca; ML; CRE	11b
Cl_a (CN9)	1	PCBR	OlG (~8 VPGs; LPOG)	SEZ; AMMC; VLP; ICL; LH; SLP	12
Cl_a (CN10)	1	PCBR	OlG (~10 PCx; DP OG)	SEZ; AMMC; VLP; PLP; LH; SIP; bCa	11c
Cl_b (CN11)	1	ACBR	OlG (VM, D OGs); EG	SLP; LH; VLP; PLP	11d
Csfs (CN12)	1	MCBR	UG (P OG); ALH	CRE; SCL; SMP; SIP; SLP	11e
Csfs (CN13)	1	MCBR	MG^a^	CRE; SCL; SMP; SIP; SLP	12
Cast (CN14)	1	ACBR	MGC‐AllGs; CL. OGs	AMMC	10c

a Full characterization was prohibited, either by co‐stained neurons, obscuring elements or weak labelling of certain neuronal compartments.

Abbreviations: ACBR, anterior cell body rind; AL, antennal lobe; ALH, antennal‐lobe hub; AMMC, antennal mechanosensory and motor center; AOTU, anterior optic tubercle; bCa, anterior base of the calyces; BL., bilateral; Ca, calyces of the mushroom bodies; Cast, centrifugal neuron in antenno‐subesophageal tract; CBU, upper division of the central body; Cl, centrifugal neuron in lateral antennal‐lobe tract; CL., contralateral; Cm, centrifugal neuron in medial antennal‐lobe tract; CRE, crepine; CSD, contralaterally projecting serotonin‐immunoreactive deutocerebral neuron; Csfs, centrifugal neuron in superior fiber system; D, dorsal; DAAR, dopaminergic arching neuron; DP, dorsoposterior; EG, extraglomerular; Gs, glomeruli; htg., heterogeneous innervations; ICL, inferior clamp; LC, antennal‐lobe lateral cell body cluster; LH, lateral horn; LPOG, labial‐palp pit organ glomerulus; MCBR, cell body rind along the midline; MG, multiglomerular; MGC‐AllGs, macroglomerular complex and all/most other glomeruli; ML, medial lobes of the mushroom bodies; OGs, ordinary glomeruli; P, posterior; PCBR, posterior cell body rind; PCx, posterior complex glomeruli; PLP, posteriorlateral protocerebrum; SCL, superior clamp; SEZ, subesophageal zone; SIP, superior intermediate protocerebrum; SLP, superior lateral protocerebrum; SMP, superior medial protocerebrum; VLP, ventrolateral protocerebrum, VPGs, ventroposterior glomeruli.

### 3.1 Antennal‐lobe projection neurons

**FIGURE 2:**
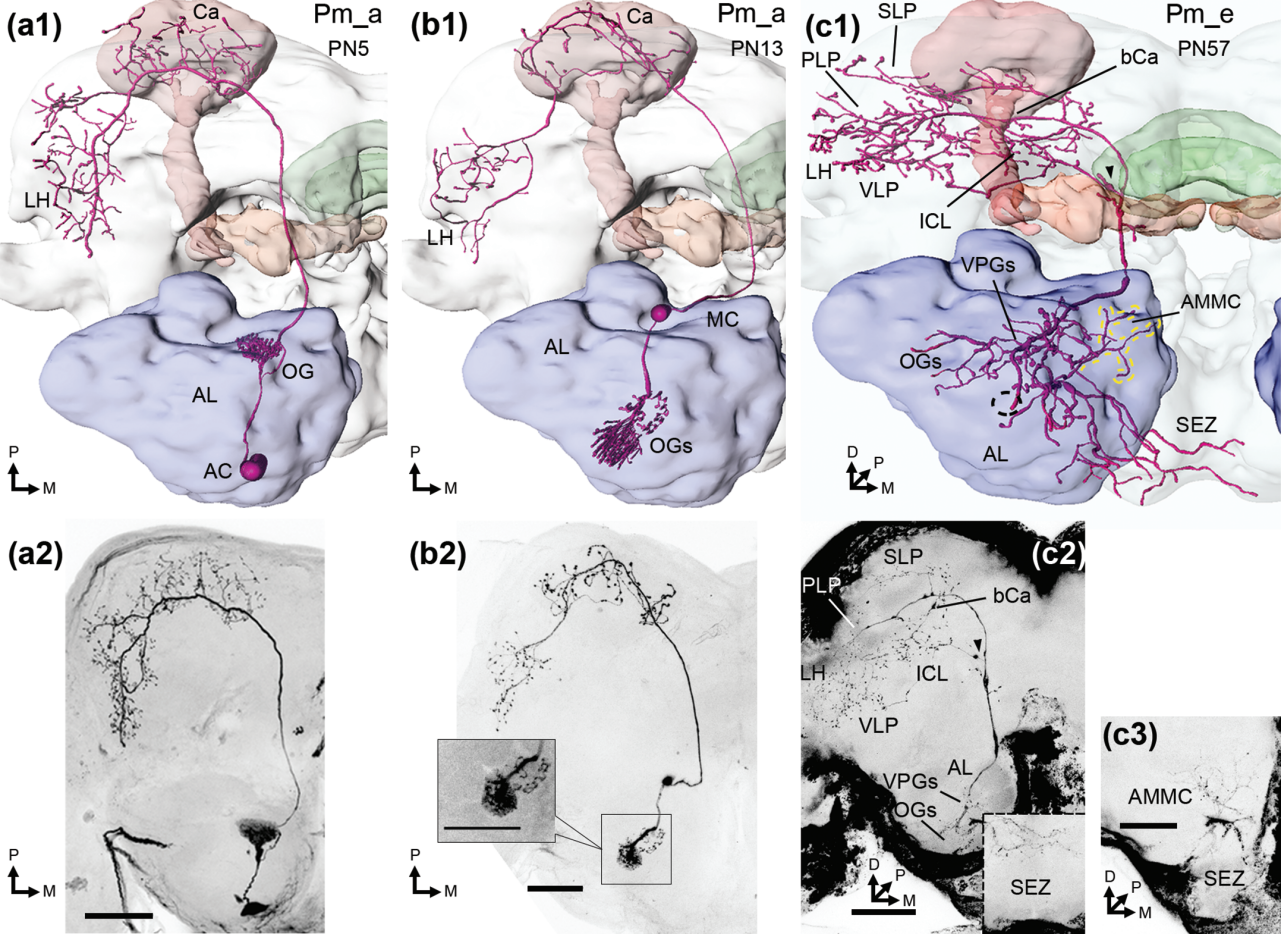
Medial‐tract PNs. (a1, a2) reconstruction and maximum intensity projection (MIP), respectively, of a uniglomerular Pm_a neuron, in dorsal view. Its cell body was in the antennal lobe (AL) anterior cell cluster (AC), while axonal targets included the calyces (Ca) and lateral horn (LH). (b1, b2) this Pm_a neuron had its soma in the medial cell cluster (MC) and projected to the Ca and LH. Its dendrites innervated two neighboring ordinary glomeruli (OGs), one densely and one sparsely, as demonstrated in the boxed single confocal section image. (c1, c2) reconstruction and two merged MIPs (dashed lines in c2) of a Pm_e neuron, with oligoglomerular dendrites in ventroposterior glomeruli (VPGs), as well as some medial and ventroanterior OGs. In addition, dendrites innervated the antennal mechanosensory and motor center (AMMC; yellow dashed lines in c1) and the subesophageal zone (SEZ; c3). The axon targeted the LH, ventrolateral protocerebrum (VLP), superior lateral protocerebrum (SLP), inferior clamp (ICL), and the base of the calyces (bCa). In addition to the main axon, a sub‐branch followed the transverse tract into the lateral protocerebrum (black arrowhead in c1 and c2). The cell body fiber led to the AC (dashed circle in reconstruction), but the soma was not visible. This PN is presented in a dorsofrontal view. D, dorsal; M, medial; P, posterior. Scale bars = 100 μm [Color figure can be viewed at wileyonlinelibrary.com]

**FIGURE 3:**
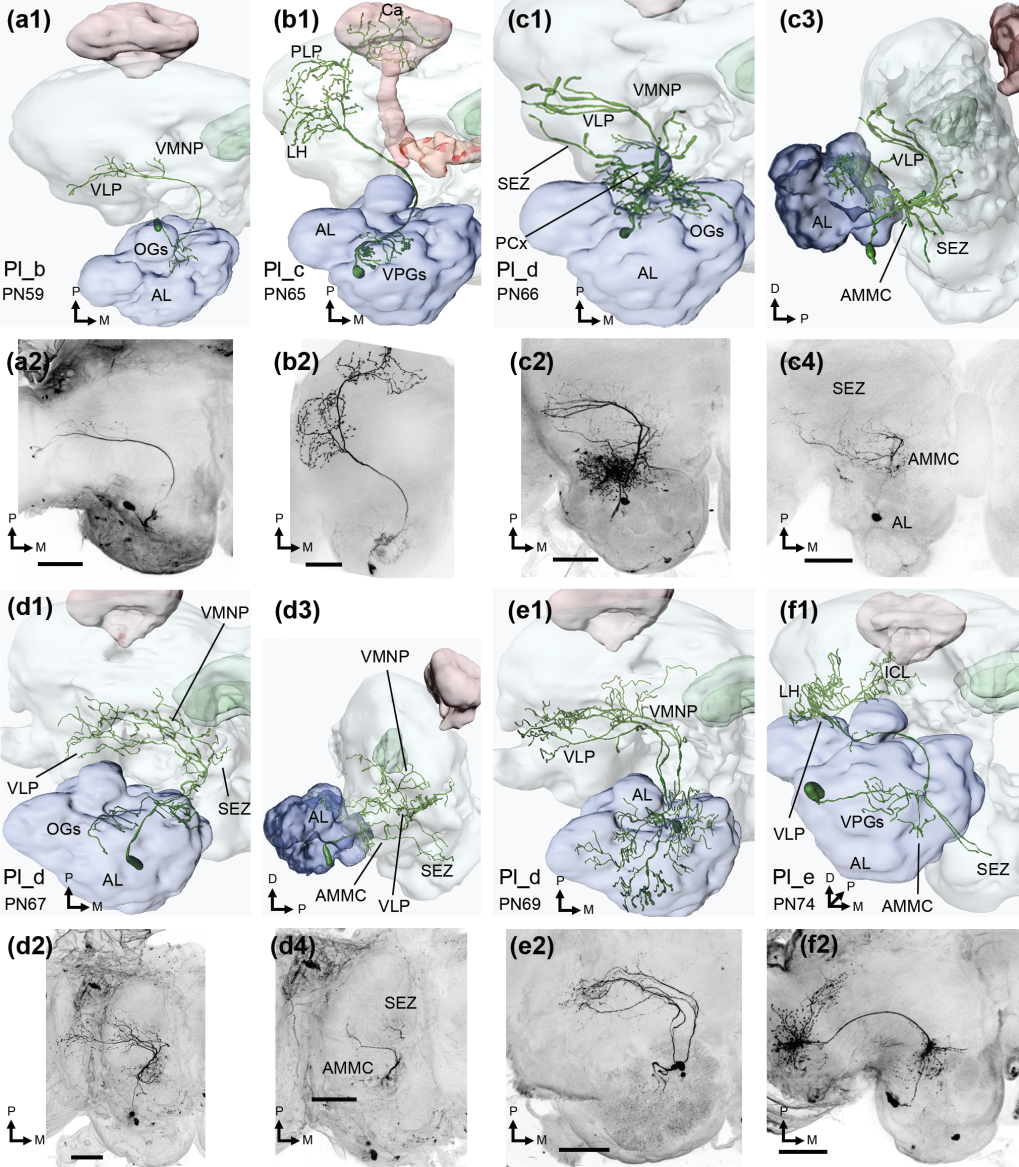
Lateral‐tract PNs. (a1, a2) reconstruction and maximum intensity projection (MIP) of an oligoglomerular Pl_b neuron with projection terminals in the ventromedial neuropil (VMNP) and ventrolateral protocerebrum (VLP). (b1, b2) a Pl_c neuron, with oligoglomerular innervation of ~six ventroposterior glomeruli (VPGs) and axonal innervation of both the lateral horn (LH) and the calyces (Ca). (c1, c2) a Pl_d neuron, which innervated posterior‐complex (PCx) glomeruli with large varicosities, and more medial ordinary glomeruli (OGs) with thin, sparse dendrites. Arborizations throughout the VMNP and VLP were also thin and smooth. In addition, the antennal mechanosensory and motor center (AMMC), and the subesophageal zone (SEZ) were innervated, as demonstrated in the sagittal reconstruction (c3) and ventral confocal stacks (c4). (d1, d2) another oligoglomerular Pl_d neuron, with projection terminals in the VLP and VMNP. Additional innervations in the AMMC and SEZ can be seen in sagittal view (d3) and a ventral MIP (d4). (e1, e2) a third Pl_d neuron, innervating all glomeruli. The axon bifurcated as it exited the antennal lobe (AL), before it forked once more at the AL isthmus. From here, the axons ran to the VLP and VMNP. (f1, f2) an oligoglomerular Pl_e neuron, innervating the VLP and LH, before turning dorsomedially to the inferior clamp (ICL; reconstruction in dorsofrontal view). This was the only Pl_e neuron innervating the AMMC and SEZ. All figures are in dorsal orientation, unless otherwise stated, and all somata were in the lateral cell cluster. D, dorsal; M, medial; P, posterior. Scale bars = 100 μm [Color figure can be viewed at wileyonlinelibrary.com]

**FIGURE 4:**
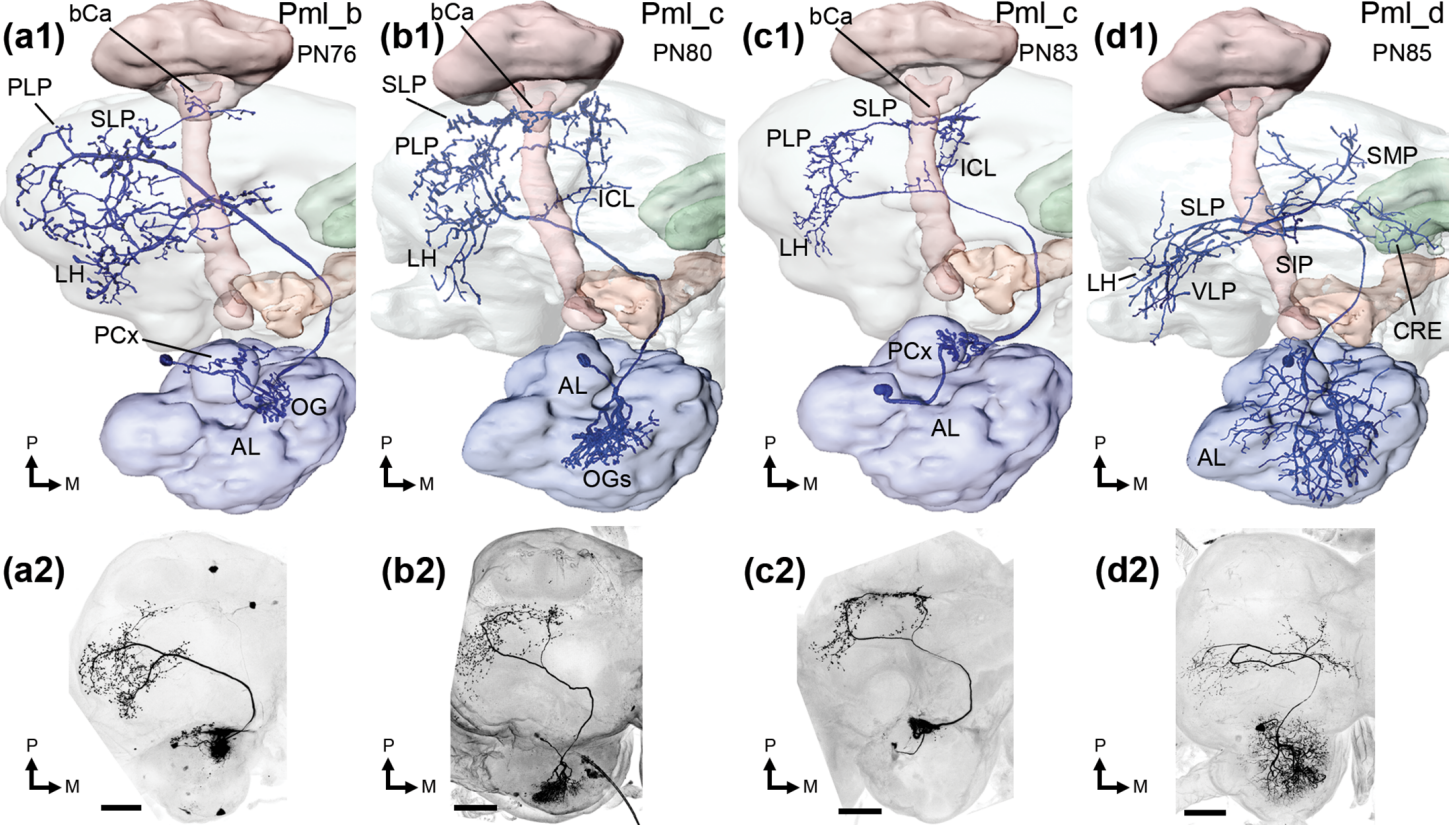
Mediolateral antennal‐lobe tract (mlALT) PNs. (a1, a2) reconstruction and maximum intensity projection of a Pml_b neuron. Antennal lobe (AL) dendrites innervated a few posterior‐complex (PCx) glomeruli and one medial ordinary glomerulus (OG). The axon projected to the lateral horn (LH), superior lateral protocerebrum (SLP), the posteriorlateral protocerebrum (PLP), and the base of the calyces (bCa). (b1, b2) an oligoglomerular Pml_c neuron, innervating a cluster of anterior OGs. Along the pathway of the mlALT, the axon bifurcated, sending the main branch to the LH, SLP, and bCa, while the thinner sub‐branch went through the inferior clamp (ICL) on its way to the bCa and SLP, where the branches converged. (c1, c2) an untypical uniglomerular Pml_c neuron, connected with G49 in PCx. (d1, d2) a Pml_d neuron, with dendrites in all glomeruli. Axonal projections included the LH, ventrolateral protocerebrum (VLP), SLP, superior intermediate and medial protocerebrum (SIP and SMP, respectively), along with the crepine (CRE). The somata of all mlALT PNs were in the lateral cell cluster, and all PNs are displayed in a dorsal orientation. M, medial; P, posterior. Scale bars in MIPs = 100 μm [Color figure can be viewed at wileyonlinelibrary.com]

**FIGURE 5:**
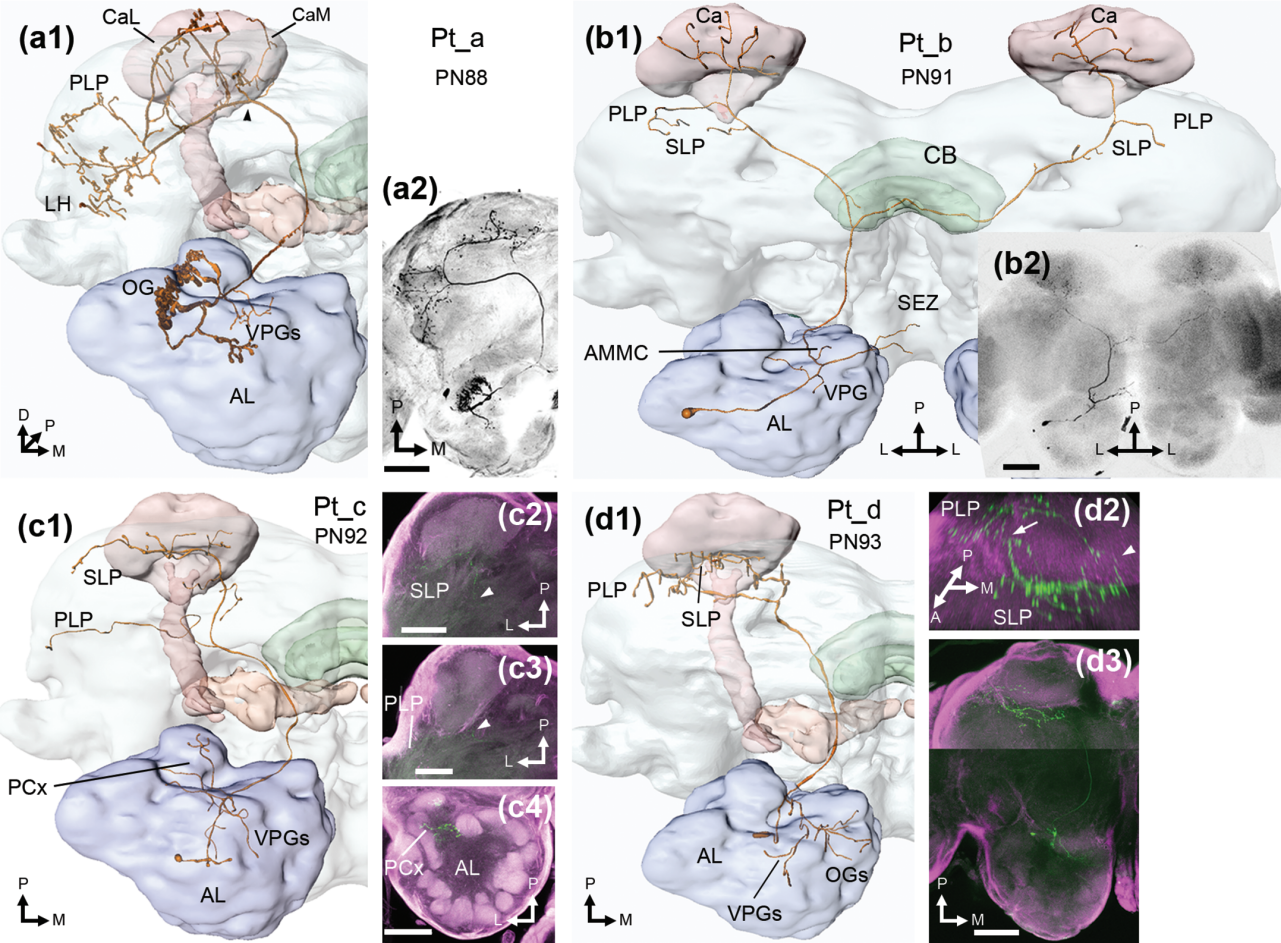
Transverse tract PNs. (a1, a2) an oligoglomerular Pt_a neuron, innervating ventroposterior glomeruli (VPGs) and an ordinary glomerulus (OG) G36. A main axon targeted the lateral horn (LH), posterior lateral protocerebrum (PLP), and both the lateral and medial calyx (CaL and CaM, respectively), while a thin sub‐branch only innervated the CaM (branching point indicated by black arrowhead). The reconstruction is in dorsofrontal view, while the maximum intensity projection (MIP) is seen dorsally. (b1, b2) a Pt_b neuron, with unilateral innervation of one VPG, G73, and some dendrites descending into the antennal mechanosensory and motor center (AMMC) and the subesophageal zone (SEZ). The bilateral axon terminated onto the calyces (Ca), superior lateral protocerebrum (SLP), and PLP. (c1) reconstruction of a faintly labeled Pt_c neuron, innervating the SLP (c2) and PLP (c3). Dendritic innervations included posterior complex (PCx) glomeruli (c4), along with some branches going to VPGs. (d1‐d3) a Pt_d neuron, branching into both VPGs and OGs. The axonal innervations are particularly evident in the tilted 3D image (d2). After the axon bifurcated (white arrowhead), one branch projected to the SLP and then the PLP, whereas the other branch went directly to the PLP, where the branches converged (white arrow). A, anterior; D, dorsal; L, lateral; M, medial; P, posterior. Scale bars = 100 μm [Color figure can be viewed at wileyonlinelibrary.com]

**FIGURE 6:**
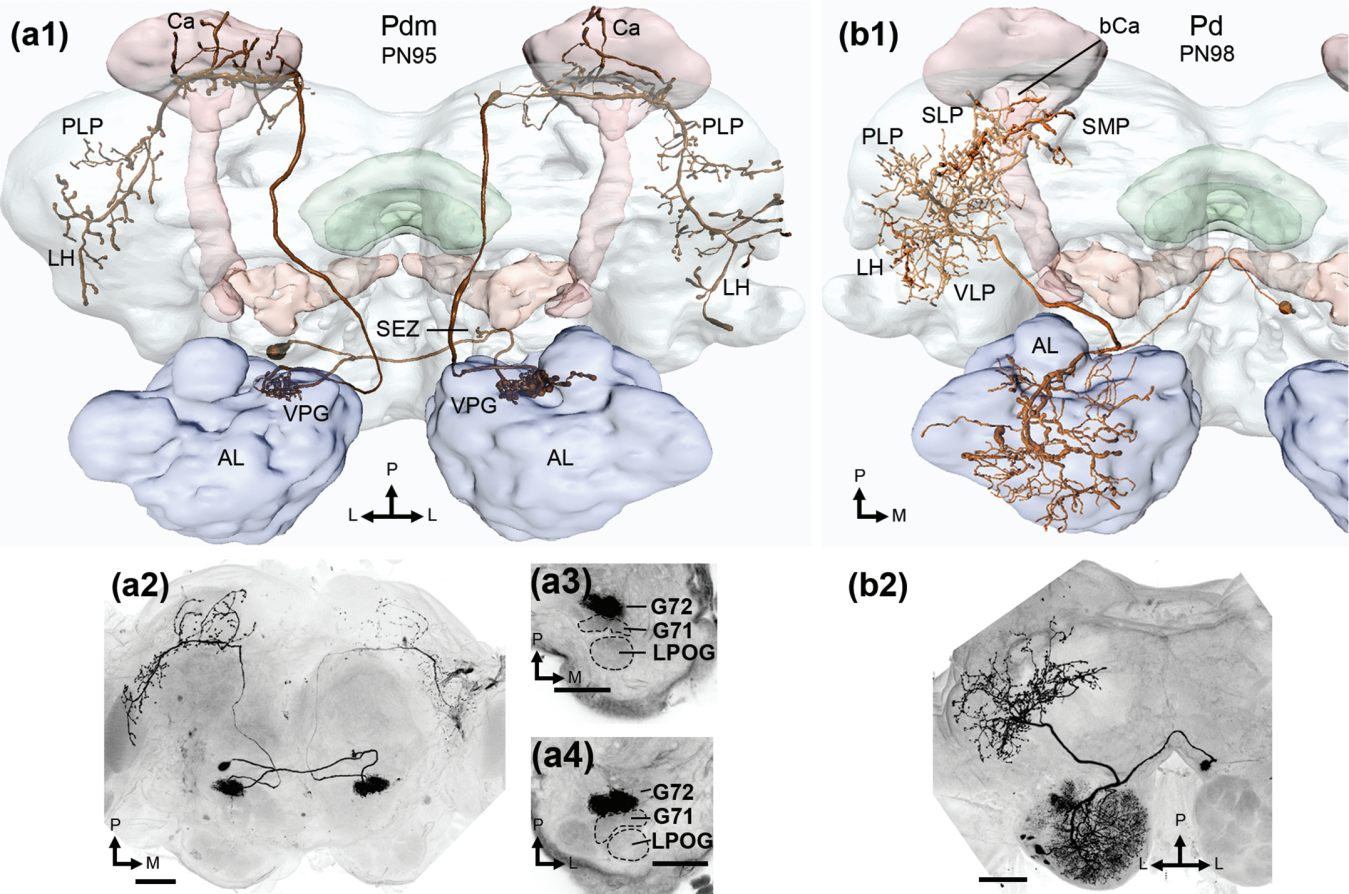
Dorsomedial‐ and dorsal‐tract PNs. (a1, a2) display of a Pdm neuron, with its soma in the subesophageal zone (SEZ), where a few processes were found. The antennal lobe (AL) glomerulus G72, that is posterior to G71 and the LPOG (a3, a4), was bilaterally innervated. The axon projected to the calyces (Ca), lateral horn (LH), and posterior lateral protocerebrum (PLP) in both hemispheres. (b1, b2) images showing the dorsal‐tract neuron, Pd. Its soma was in ventral parts of the medial cell cluster, on the contralateral side to the dendrites and axon. While dendrites innervating many glomeruli can be seen, a full account of the neuron's arborizations was prohibited due to co‐staining of a local interneuron. The PN's axon innervated the ventrolateral protocerebrum (VLP), LH, PLP, the superior lateral and medial protocerebrum (SLP and SMP, respectively). All images in dorsal orientation. L, lateral; M, medial; P, posterior. Scale bars = 100 μm [Color figure can be viewed at wileyonlinelibrary.com]

**FIGURE 7:**
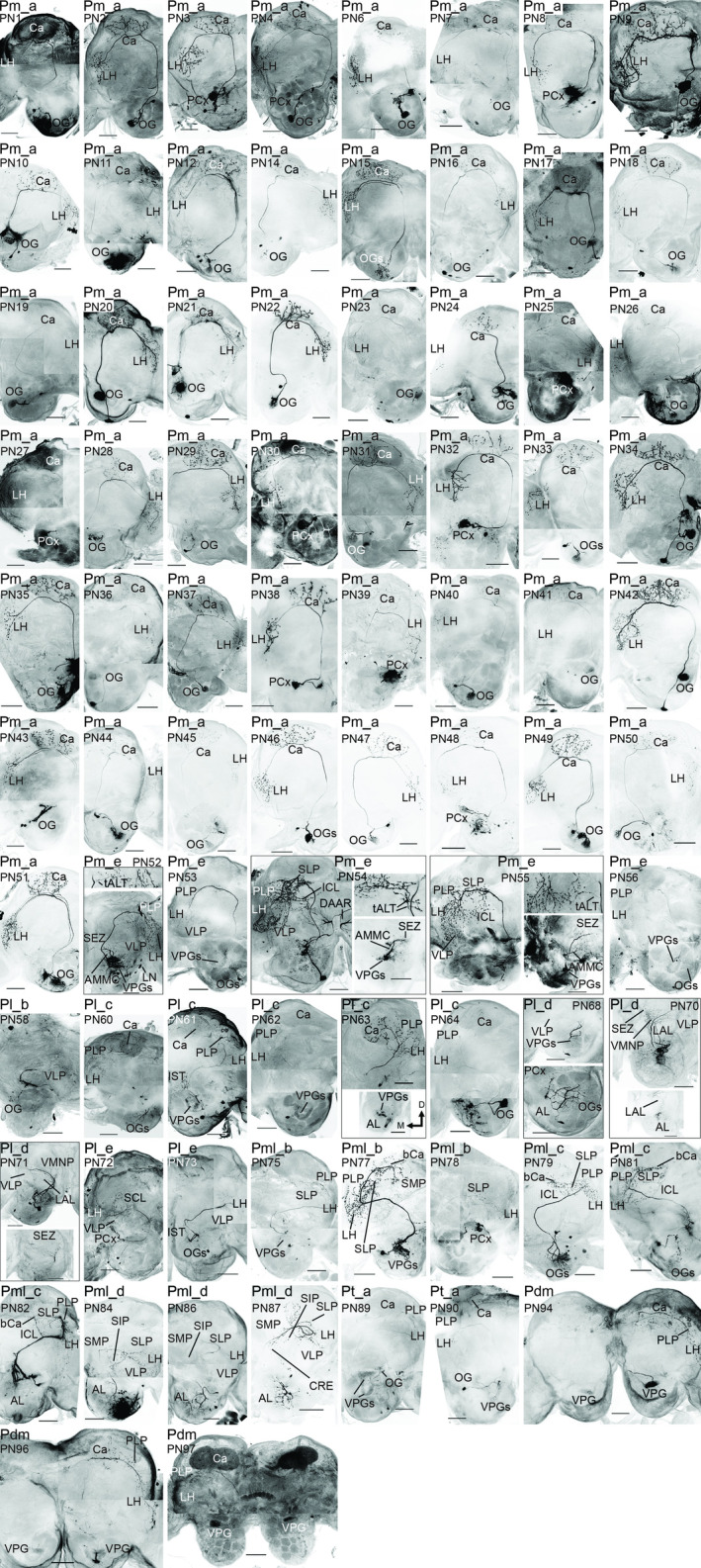
Confocal images of PNs. These images include all individual PNs not shown in Figures 2, 3, 4, 5, 6. Where necessary, two or more maximum intensity projections were merged. AL, antennal lobe; AMMC, antennal mechanosensory and motor center; bCa, anterior base of the calyces; Ca, calyces; CRE, crepine; DAAR, dopaminergic arching neuron; ICL, inferior clamp; IST, AL isthmus; LAL, lateral accessory lobe; LH, lateral horn; LN, local interneuron; OG, ordinary glomerulus; PCx, posterior complex glomeruli; PLP, posterior lateral protocerebrum; SCL, superior clamp; SEZ, subesophageal zone; SIP, superior intermediate protocerebrum; SLP, superior lateral protocerebrum; SMP, superior medial protocerebrum; tALT, transverse ALT; VLP, ventrolateral protocerebrum; VMNP, ventromedial neuropil; VPG; ventroposterior glomerulus. Scale bars = 100 μm

A PN connects the AL and other brain regions via one of six ALTs. We managed to stain PNs in every ALT: 67 in the medial ALT, 18 in the lateral ALT, 13 in the mediolateral ALT, 6 in the transverse ALT, 4 in the dorsomedial ALT, and 1 in the dorsal ALT (Table 2; Figure 2, 3, 4, 5, 6, 7). We classified these 109 PNs into different types according to the ALT the axon projected through and divided each PN type into distinct sub‐types based primarily on axonal projection patterns in the protocerebrum. For clarity, for the Pm_a neurons, *P* indicates that the category is PN, *m* applies to the medial‐tract type, while *a* refers to the sub‐type. This naming system is an adaptation of the terms introduced by Homberg et al. (1988).

#### 3.1.1 Projection neurons in the medial antennal‐lobe tract

In total, 67 mALT neurons were stained across 57 preparations. As many as 61 of these PNs were classified as Pm_a sub‐type, that is largely homogenous uniglomerular PNs targeting the calyces and LH (Figure 2a;Homberg et al., 1988; Ian, Zhao, et al., 2016). These PNs had somata in all three AL cell clusters, with 38% in the LC, 44% in the MC, and 18% in the AC. All reported Pm_a neurons were restricted to OGs or PCx glomeruli, and had comparable protocerebral innervations. However, some PCx PNs innervated more dorsomedial parts of the LH than the OG PNs (e.g., PN3; Figure 7). Unlike most previous reports, we found that a few Pm_a neurons were not uniglomerular. Four Pm_a neurons innervated two OGs—filling one OG densely, and the outer layer of another OG more sparsely (Figure 2b). Furthermore, another two co‐stained Pm_a neurons (PN15; Figure 7) collectively innervated six OGs.

Six neurons of the Pm_e sub‐type were labeled. As previously reported by Ian, Zhao, et al. (2016), these PNs project terminal branches into several protocerebral neuropils, including the LH, ventrolateral protocerebrum (VLP), posteriorlateral protocerebrum (PLP), superior lateral protocerebrum (SLP), and the inferior clamp (ICL)—but not in the cups of the calyces. In addition to the main axon, four PNs had a sub‐branch that followed the tALT towards the lateral protocerebrum (PNs 52, 54–55, 57; Figure 2c and 7). These four PNs also innervated the subesophageal zone (SEZ) and the AMMC. The Pm_e neurons had oligoglomerular dendrites in ventral glomeruli, including VPGs and OGs. Some of these PNs were co‐stained with LNs, therefore only traceable dendrites were considered as part of the Pm_e neurons.

#### 3.1.2 Projection neurons in the lateral antennal‐lobe tract

Altogether 18 PNs in the lALT were stained across 17 preparations; these consisted of four distinct sub‐types. All lALT PNs had somata in the LC, while dendritic and axonal innervations differed across and within most sub‐types, confirming the previously reported heterogeneity of neurons in this tract (Homberg et al., 1988; Ian, Zhao, et al., 2016).

Two Pl_b neurons were labeled, both terminated primarily in the VLP. One PN had pronounced club‐like terminals (Figure 3a), as reported previously in *H. virescens* (Ian, Zhao, et al., 2016) and *M. sexta* (Homberg et al., 1988). The two neurons' dendritic innervation patterns differed, as one was uniglomerular while the other was oligoglomerular.

Six Pl_c neurons were identified (Figure 3b). These PNs terminate broadly in the LH and PLP, before targeting the calyces (Hansson et al., 1994; Homberg et al., 1988; Namiki & Kanzaki, 2011; Rø et al., 2007). One Pl_c neuron had uniglomerular innervations in an OG, while the remaining five had varying extents of oligoglomerular arborizations. Four of these PNs (PNs 61–63 and 65) had identical dendritic patterns, linked to the VPGs G64‐G69.

The widely projecting Pl_d sub‐type, first reported by Homberg et al. (1988), included six stained PNs. All of them targeted the ventromedial neuropil (VMNP) and VLP, but their additional innervations differed. Three of the Pl_d neurons, PNs 66–68, were oligoglomerular, and further innervations included the SEZ and AMMC (Figure 3c,d). The remaining three, PNs 69–71, arborized in all glomeruli (Figure 3e), with additional innervations including the SEZ and lateral accessory lobe (LAL; Figure 7). One of the first‐mentioned, oligoglomerular neurons, PN66, had heterogeneous arborizations in the AL including smooth dendrite‐like processes in a few OGs and thick projections with varicosities in the PCx glomeruli.

The Pl_e sub‐type (Ian, Zhao, et al., 2016) comprised four stained neurons in three preparations. Axonal targets commonly included the VLP, LH, and either the ICL or the superior clamp (SCL), while one neuron (PN74; Figure 2f) also innervated the SEZ and AMMC. The dendritic arborizations of this PN sub‐type were uni‐ or oligo‐glomerular.

#### 3.1.3 Projection neurons in the mediolateral antennal‐lobe tract

Thirteen mlALT PNs were stained. All passed along the initial part of the mALT, then bent laterally at the anterior edge of the central body, running towards the lateral protocerebrum. All Pml neurons had their somata in the LC, and with one exception, all had multiglomerular dendritic innervations, which were quite consistent within sub‐types. In addition to the previously reported Pml_b (Ian, Zhao, et al., 2016), we present two additional sub‐types.

The Pml_b sub‐type included four stained PNs sending their axon to the LH and surrounding neuropils, including the PLP, SLP and the anterior base of the calyces (bCa; Figure 4a). In addition, PN75 and PN77 innervated regions along the border of the SCL and the superior medial protocerebrum (SMP). All Pml_b neurons were oligoglomerular, with dendrites limited to at most four heterogeneously innervated VPGs or PCx glomeruli.

Five labeled mlALT PNs were found to constitute a novel sub‐type, which we termed Pml_c. A prominent Y‐shaped bifurcation of the axon, located ventrally to the pedunculus, typified the Pml_c neurons. From here, the main fiber targeted the LH and PLP, before turning dorsomedially to the SLP and bCa. In addition, a thin sub‐branch projected through the ICL on its way to the bCa, where it converged with the other branch. Three Pml_c neurons had oligoglomerular innervations in a cluster of anteriorly located OGs (Figure 4b), while one was uniglomerular (Figure 4c), innervating the PCx glomerulus G49. Only one uniglomerular mlALT PN has previously been reported in moths (Namiki & Kanzaki, 2011). The last Pml_c neuron had weakly stained dendrites, preventing detailed characterization.

The last sub‐type, Pml_d, included four stained PNs characterized by their unique projection path. One typical Pml_d neuron (PN85) is presented in Figure 4d; after projecting through the VLP and LH, its axon made a U‐turn and passed on dorsomedially to the SLP and the superior intermediate protocerebrum (SIP), before finally ending up in the crepine and SMP, close to the brain midline. As demonstrated, the PN extended terminal branches along its entire course. In the AL, the neuron arborized extensively with dendrites in most or all glomeruli. The dendritic morphology of all Pml_d neurons corresponded to PN85. A similar PN has previously been reported in *M. sexta* (see Figure 8c in Homberg et al., 1988).

**FIGURE 8:**
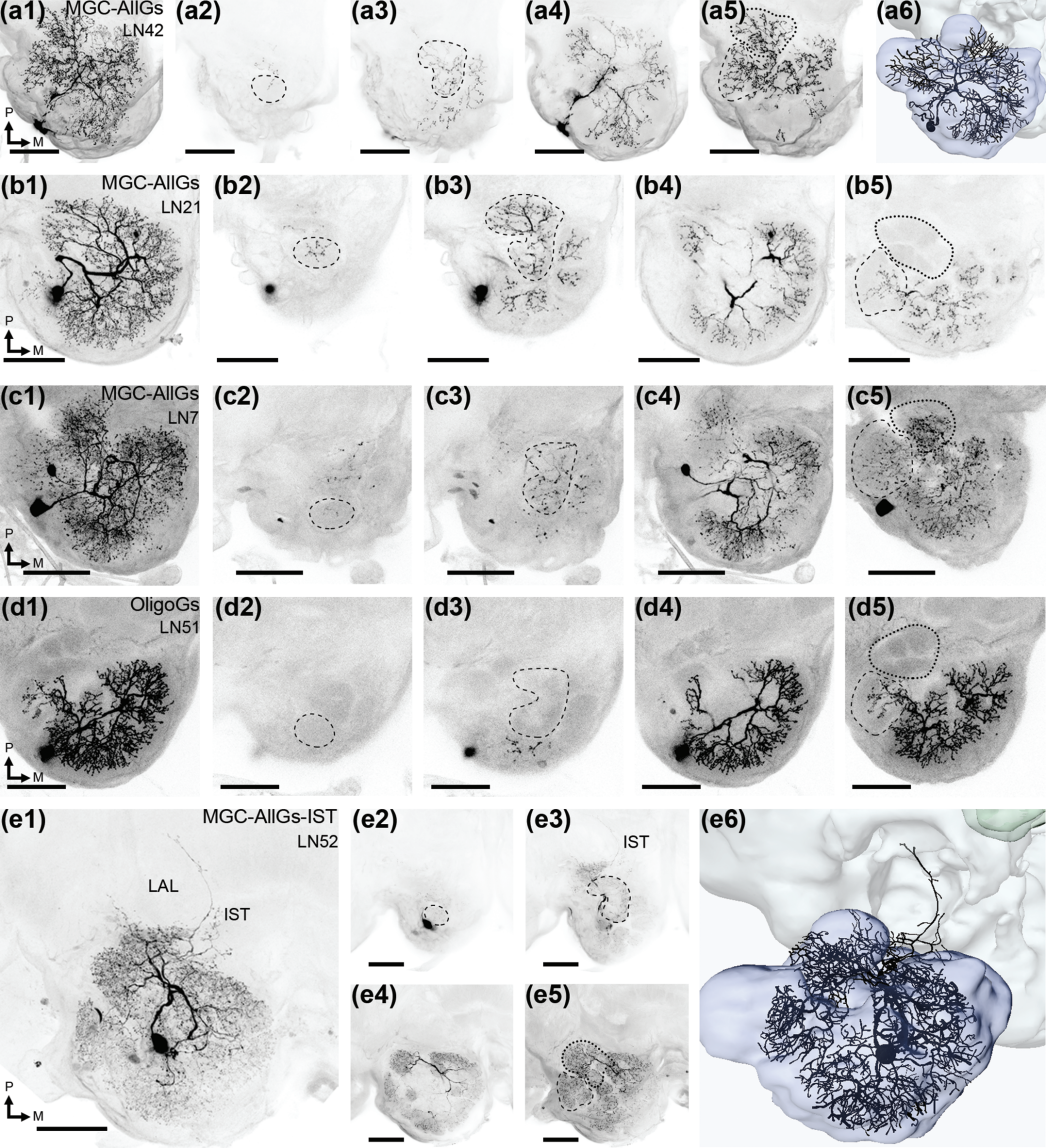
Antennal‐lobe (AL) LNs. An MGC‐AllGs LN is presented in a1‐a6. A maximum intensity projection of the confocal stacks covering the entire AL can be seen in a1, while the following images demonstrate selected regions of the AL, with specific glomerular sub‐systems indicated by dashed lines. The labial palp‐pit organ glomerulus (LPOG) was sparsely innervated (a2), while the ventroposterior glomeruli (VPGs; a3) included more processes. As in all MGC‐AllGs LNs, the ordinary glomeruli (OGs; a4) were substantially innervated. This particular LN also innervated the macroglomerular complex and posterior complex (MGC and PCx; dashed and dotted lines in a5, respectively). In a6, a reconstruction of this LN is presented. Marked glomeruli in b‐e are arranged like a1‐a6. (b1‐b5) an MGC‐AllGs LN with sparse innervation of the MGC, and no innervations in the PCx glomeruli. (c1‐c5) two co‐stained MGC‐AllGs LNs, with sparse dendrites in the LPOG and VPGs. (d1‐d5) an oligoglomerular LN, which had weak innervation of the MGC, and no innervations of the LPOG, VPGs or PCx glomeruli. (e1‐e6) one of the MGC‐AllGs‐IST type LNs, stretching outside the AL to the proximal AL isthmus (IST), and surrounding the lateral accessory lobe (LAL). These LNs filled all glomeruli. M, medial; P, posterior. Scale bars = 100 μm [Color figure can be viewed at wileyonlinelibrary.com]

#### 3.1.4 Projection neurons in the transverse antennal‐lobe tract

Six tALT neurons were labeled, including one bilateral PN. These PNs formed four sub‐types, all following the mALT before bending laterally at the posterior border of the central body, between the turning points of the m‐ and ml‐ALTs. The axons of all unilateral tALT neurons bifurcated when leaving the mALT, and both sub‐branches often converged onto the same neuropil. The PNs we classify as Pt_a and Pt_d have previously been reported as mALT PNs, that is as Pm_c and Pm_b sub‐types, respectively (Homberg et al., 1988; Rø et al., 2007).

The most prevalent tALT sub‐type, which we named Pt_a (Figure 5a), included three labeled PNs. Axonal targets were limited to the LH, PLP, and the calyces, where the medial calyx was innervated by both the thick‐ and thin sub‐branches. All Pt_a neurons arborized in similar AL glomeruli, including the VPGs (G64‐G69) and the large posterior OG, G36.

We also present a bilateral Pt_b sub‐type (Figure 5b), comprising one neuron with smooth branches in the SEZ, AMMC, and the VPG, G73. From the ventroposterior side of the central body, the axon split, sending one branch into the contralateral hemisphere. The PN extended small, sparsely distributed terminals into the calyces, PLP, and SLP of both hemispheres.

The third sub‐type, named Pt_c, included one faintly labeled, very thin PN (Figure 5c). Axonal projections included the PLP and SLP, while dendritic branches innervated three PCx glomeruli, and included thin arborizations in the VPG region. Another PN constituted a fourth sub‐type, termed Pt_d (Figure 5d). From the bifurcation point, one process ran to the PLP and SLP, whereas the other branch projected directly into the SLP, where the branches converged. Each branch extended terminals along their routes. The oligoglomerular PN extended thin and highly branched dendrites in the AL, innervating medial OGs and VPGs.

#### 3.1.5 Projection neurons in the dorsomedial and dorsal antennal‐lobe tracts

Four bilateral dmALT PNs were stained (Figure 6a). These Pdm neurons were morphologically homogenous (Figure 7), and like bilateral PNs previously reported in *H. virescens* (Ian, Zhao, et al., 2016; Rø et al., 2007). Here, we present new details on the neurons' axonal and dendritic innervations in both hemispheres. At least two of four Pdm neurons had cell bodies in the cell body rind lateral to the gnathal ganglion, that is in the SEZ. From here, a neurite projected dorsally and bifurcated, densely filling a single mirror‐symmetric glomerulus in each AL. All Pdm neurons had dendrites exclusively in VPGs; two PNs innervated G71, while the other two innervated G72. In addition, one Pdm neuron had a few varicosities in the SEZ. In each hemisphere, the axon exiting the AL passed the central body dorsally. Terminal projections permeated the calyces and finally terminated broadly in the LH, along with the PLP. The lateral protocerebral terminations appeared uniform in both hemispheres.

We present one dALT PN (Figure 6b), similar to a PN reported in *M. sexta* (Kanzaki et al., 1989). The Pd neuron's soma was localized in the ventral part of the MC in the contralateral hemisphere. From the soma, the main neurite crossed the brain midline through the inferior AL commissure, before bifurcating at the AL border. From here, one branch entered the AL, while another projected to widespread protocerebral neuropils. Axon terminals were found in the VLP, PLP, LH, SLP, and the base of the calyces, as well as the border of the SIP and SMP. A co‐stained LN prevented full AL characterization, but the thick dendritic branch appeared to innervate most glomeruli, excluding the MGC.

### 3.2 Antennal‐lobe local interneurons

**FIGURE 9:**
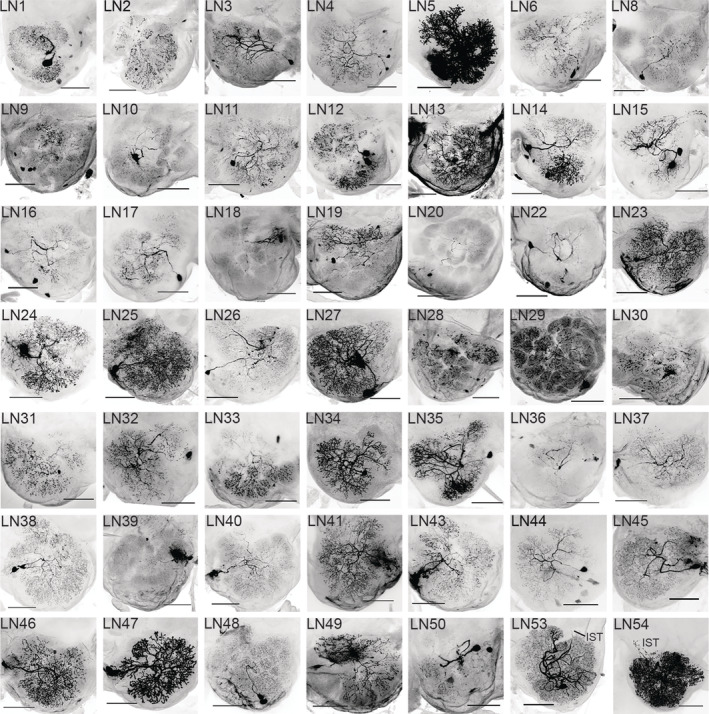
Confocal images of LNs. These neurons cover all the LNs not shown in Figure 8. LN1‐48 are MGC‐AllGs type neurons, while LN49 and 50 are oligoglomerular neurons. Finally, LN53 and 54 belong to the MGC‐AllGs‐IST type, stretching outside the antennal‐lobe into the isthmus (IST). Scale bars = 100 μm

Altogether, 67 LNs were stained across 54 preparations (Table 3; Figures 8 and 9). These LNs comprised three types, differentiated on the background of dendritic innervation patterns. The most frequently stained LNs were the MGC‐AllGs LNs (Seki & Kanzaki, 2008), which innervate all or most glomeruli (Figure 8a). In total, 60 MGC‐AllGs LNs were stained across 48 preparations, all innervating most OGs quite homogenously. However, innervations often varied across AL sub‐systems, as all glomerular groups except for the OGs were sparsely innervated by some MGC‐AllGs LNs. Most notably, 30% had sparse innervation of the MGC (Figure 8b). In the PCx, 18% had sparse innervation, 2% particularly dense innervation, and 2% no innervation at all (Figure 8b). As for the LPOG, 17% had sparse innervations and 9% no innervation. Finally, for the VPGs, 14% had sparse innervations (Figure 8c) and 5% dense innervation of some glomeruli, especially those immediately posterior to the LPOG.

The second LN type included three oligoglomerular neurons. These OligoGs LNs innervated ~60–70% of the glomeruli. One example is LN49, innervating most OGs, VPGs, and the LPOG, but not the PCx or MGC. Conversely, LN51 had sparse MGC innervations, and normal arborization in many OGs, but no dendrites in the VPGs, PCx, and LPOG (Figure 8d).

A third and novel type of LN, called MGC‐AllGs‐IST, comprised four neurons across three preparations. In addition to innervating all glomeruli, these LNs extended just outside the ventroposterior AL into the AL isthmus, that is a region immediately lateral to the root of the lALT, where processes of lALT PNs have previously been reported (Homberg et al., 1988). Furthermore, LN52 (Figure 8e) enveloped the LAL through two thin branches. No branches exiting the AL appeared to be axons, determined by their thinness and lack of obvious axon terminals, and none of the LNs projected onward from the AL‐proximal parts to terminate onto additional neuropils.

### 3.3 Antennal‐lobe centrifugal neurons

**FIGURE 10:**
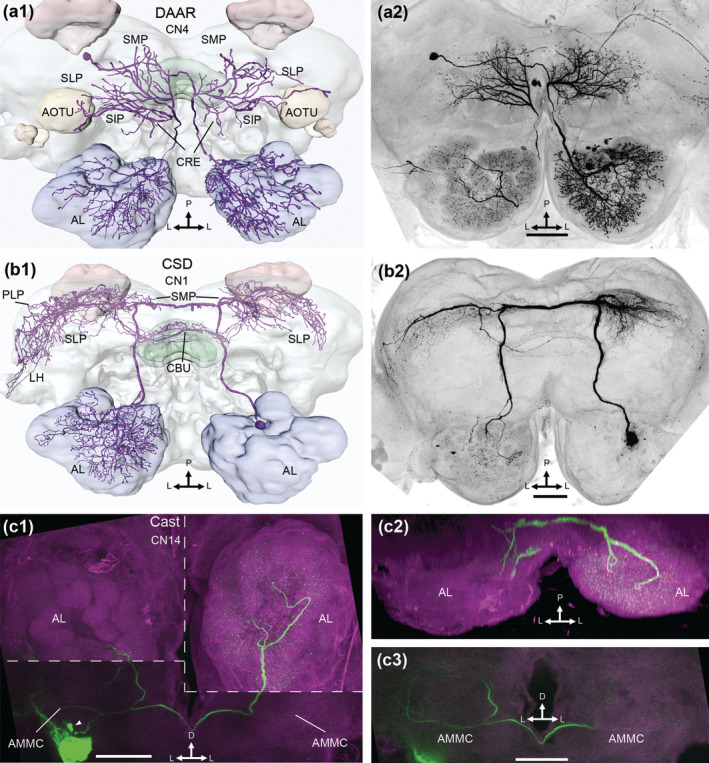
Bilateral CNs. (a1, a2) reconstruction and maximum intensity projection (MIP) displaying the dopaminergic arching neuron (DAAR) in a dorsal view. The soma was in the posterior cell body rind. The bilateral dendrites innervated the superior medial, intermediate, and lateral protocerebrum (SMP, SIP, and SLP, respectively), the crepine (CRE), and the anterior optic tubercle (AOTU). Axons terminated broadly in the antennal lobes (ALs), but the density of innervation in each AL differed. (b1, b2) the contralaterally projecting serotonin‐immunoreactive deutocerebral (CSD) neuron, with its soma in the lateral cell cluster of the AL. The SMP, SLP, and the upper division of the central body (CBU) were bilaterally innervated, while the lateral horn (LH) and AL was only contralaterally innervated. (c1) three merged MIPs (white dotted lines) showing the Cast neuron in frontal view, with bilateral asymmetric innervations of both the antennal mechanosensory and motor centers (AMMCs) and the ALs. The soma (white arrowhead, just dorsal to a dye leakage) was in the anterior cell body rind. (c2) a 3D rendering of the AL innervations, in dorsal view. The axons travelled along the antenno‐subesophageal tracts and entered each AL from the ventroposterior side. There, contralateral innervations filled all glomeruli, whereas the ipsilateral AL branches were mostly in ventroposterior regions. (c3) MIP demonstrating the bilateral AMMC innervations. D, dorsal; L, lateral; P, posterior. Scale bars = 100 μm [Color figure can be viewed at wileyonlinelibrary.com]

**FIGURE 11:**
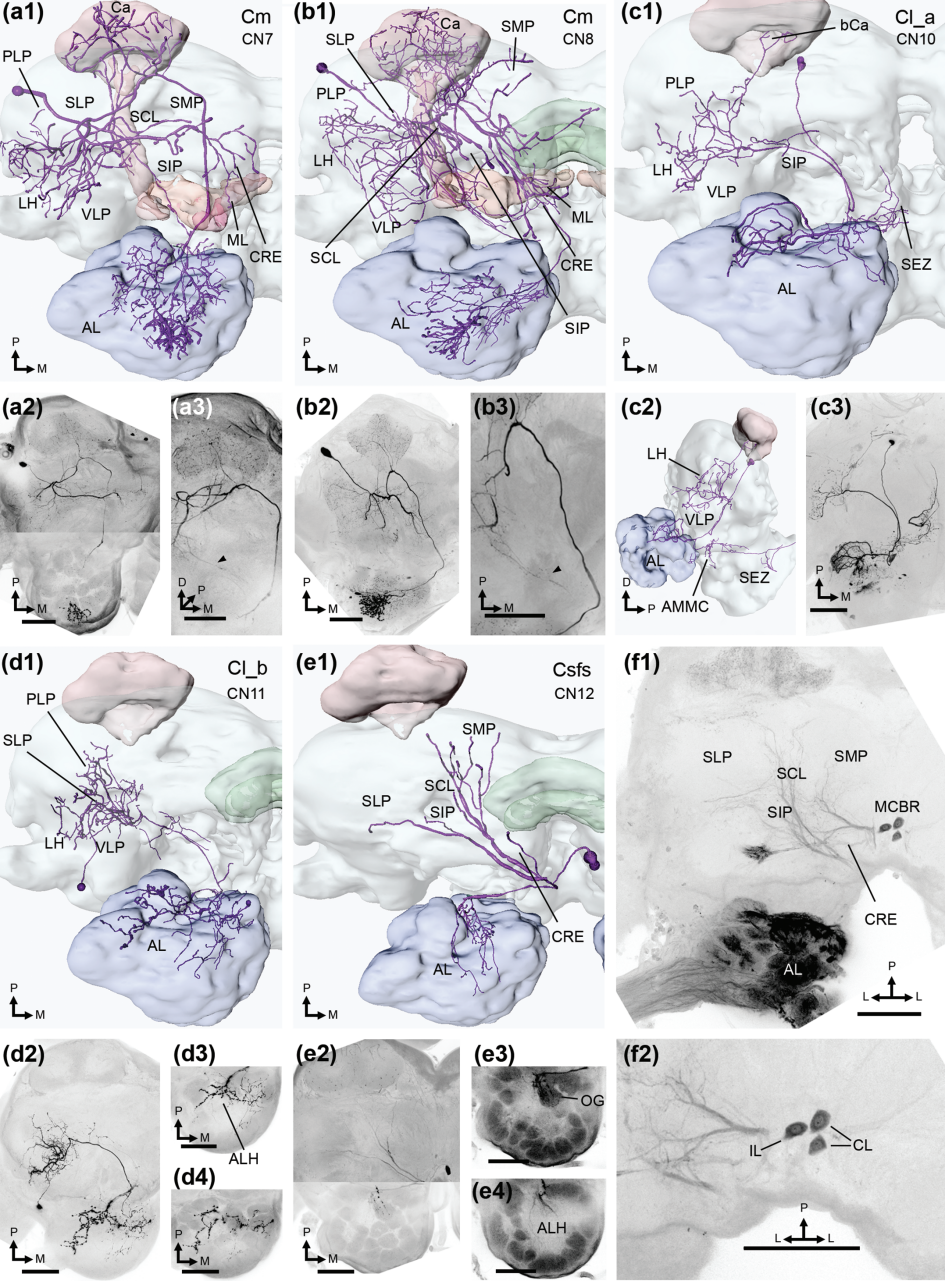
Unilateral CNs. (a1, a2) a Cm neuron, with its soma in the posterior cell body rind (PCBR). Dendrites branched through the posteriorlateral protocerebrum (PLP), lateral horn (LH), ventrolateral protocerebrum (VLP), superior clamp (SCL), and the superior lateral, intermediate, and medial protocerebrum (SLP, SIP, and SMP, respectively). In addition, the medial lobes (ML), the adjacent crepine (CRE), and the calyces (Ca) were innervated. An axon projected to the antennal lobe (AL) through the medial antennal lobe tract (mALT). All AL glomeruli were filled, but a cluster of anterior ordinary glomeruli (OGs) received particularly large terminals. (a3) Dorsofrontal image displaying branches following both the mALT and the lateral ALT (lALT; black arrowhead). (b1‐b3) another Cm neuron, sampled from a female moth. The two Cm neurons were largely similar, including the sub‐branch in the lALT (black arrowhead in b3). (c1‐c3) a female moth's Cl_a neuron, with its soma in the PCBR. The dendrites innervated the VLP, LH, PLP, SIP, and the base of the calyces (bCa). The axon followed the lALT to the AL, where approximately 10 dorsoposterior glomeruli were innervated. The neurite then projected ventrally into the antennal mechanosensory and motor center (AMMC) and the subesophageal zone (SEZ; see sagittal view in c2). (d1, d2) the Cl_b neuron, which had its soma in the anterior cell body rind. Dendrites innervated the VLP, LH, PLP and SLP. The axon followed the lALT into the AL, where medial OGs were innervated with small processes, while another branch sent large varicosities into the antennal lobe hub (ALH; d3). In addition, the latter branch terminated in the dorsal AL, primarily in extraglomerular spaces (d4). (e1, e2) a Csfs neuron, following the superior fiber system (SFS). The CN's soma was in the cell body rind along the midline (MCBR), contralateral to all innervations. The dendrites ran through the CRE, SIP, SLP, SCL, and SMP. The AL processes included innervation of one dorsoposterior OG (e3), along with branches in the ALH (e4). (f1, f2) dorsal confocal stacks from a mass staining of an AL demonstrated three labeled MCBR somata, along with several branches following the SFS. Two somata were located contralaterally (CL) to the labeled AL, while one was in the ipsilateral hemisphere (IL). All figures are in dorsal orientation, unless otherwise stated. D, dorsal; L, lateral; M, medial; P, posterior. Scale bars = 100 μm [Color figure can be viewed at wileyonlinelibrary.com]

**FIGURE 12:**
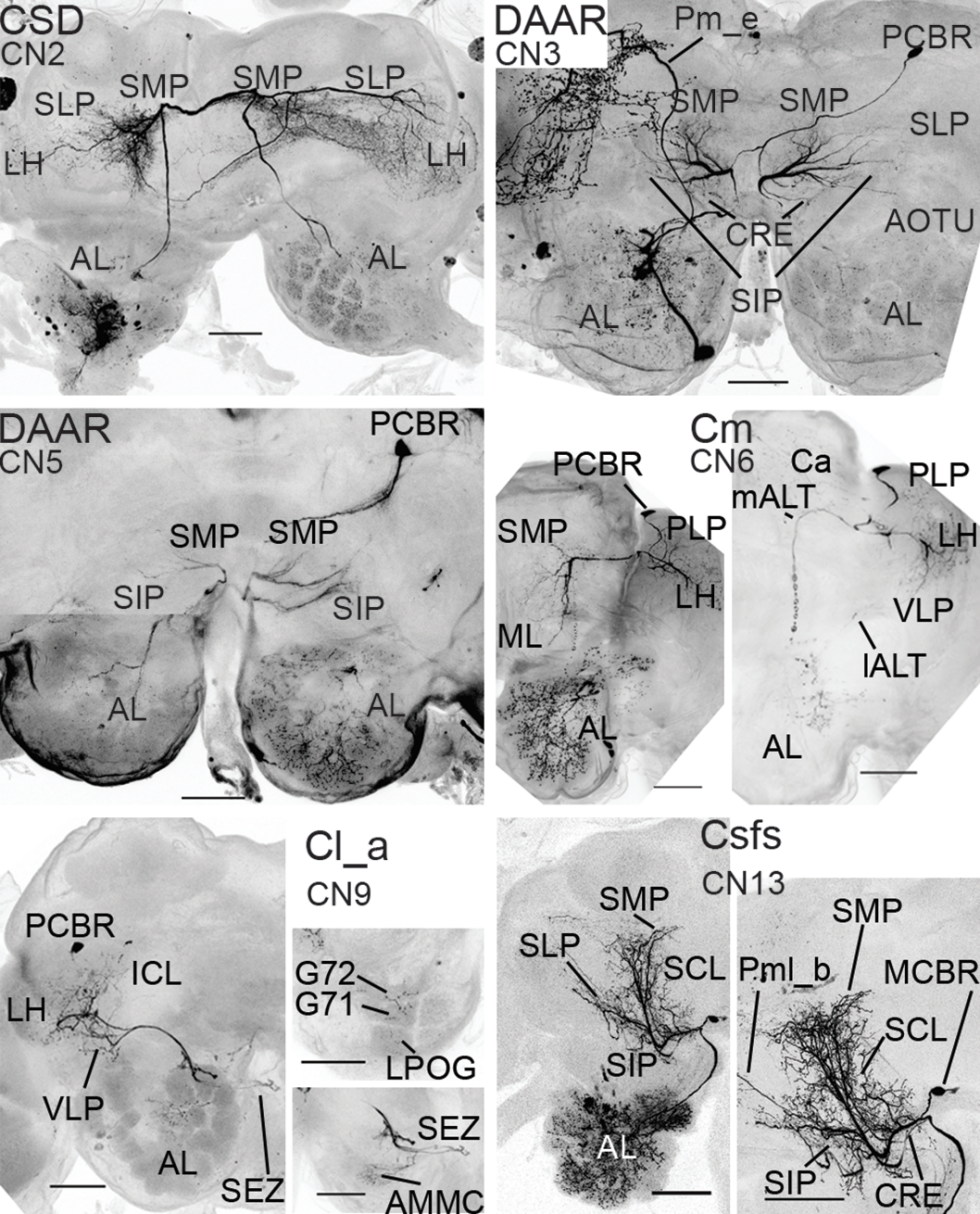
Confocal images of CNs. These images include all CNs not displayed in Figures 10 & 11. Where necessary, two or more maximum intensity projections were merged. AL, antennal lobe; AMMC, antennal mechanosensory and motor center; AOTU, anterior optic tubercle; Ca, calyces; CRE, crepine; CSD, contralaterally projecting serotonin‐immunoreactive deutocerebral neuron; DAAR, dopaminergic arching neuron; ICL, inferior clamp; lALT, lateral antennal‐lobe tract; LH, lateral horn; LPOG, labial palp‐pit organ glomerulus; mALT, medial antennal‐lobe tract; MCBR, medial cell body rind; ML, medial lobes of the mushroom body; PCBR, posterior cell body rind; PLP, posterior lateral protocerebrum; SCL, superior clamp; SEZ, subesophageal zone; SIP, superior intermediate protocerebrum; SLP, superior lateral protocerebrum; SMP, superior medial protocerebrum; VLP, ventrolateral protocerebrum. Scale bars = 100 μm [Color figure can be viewed at wileyonlinelibrary.com]

Fourteen labeled AL CNs formed seven distinct types (Table 4; Figures 10, 11, 12), of which at least three are novel. Among the CNs, six were bilateral, while the remaining eight were generally constricted to one hemisphere.

#### 3.3.1 Bilateral centrifugal neurons

Three types of bilateral CNs were stained. The first type included three CNs categorized as dopaminergic arching (DAAR) neurons (Figure 10a), based on morphological similarity to a CN described in *M. sexta* (Dacks et al., 2012; Lizbinski et al., 2016). The CN's soma was in the posterior cell body rind, ventrally to the pedunculus. From here, the primary neurite extended medially and bifurcated dorsally to the central body, each neurite giving rise to bilaterally symmetric dendritic fields, including dense innervations in the SMP, crepine, and SIP, along with sparse dendrites in the SLP and the anterior optic tubercle (AOTU). The two neurites then ran ventroanteriorly, entering the AL ventromedially to the mALT. In each AL, most glomeruli were innervated by blebby terminals, however the size and density of these terminals appeared to be bilaterally asymmetric.

The second bilateral CN type was the CSDn (Figure 10b), which has been examined in many insect orders (reviewed by Dacks, Christensen, & Hildebrand, 2006). The somata of the two CSD neurons stained here were in the AL's LC. From here, a thick neurite projected through the ipsilateral mALT to dendritic fields that covered large parts of the ipsi‐ and contralateral SMP, SLP, PLP, and LH, as well as the upper division of the central body. After projecting along the mALT in the contralateral hemisphere, all glomeruli of the contralateral AL were targeted.

A third bilateral CN type included one CN with its soma in the anterior cell body rind, ventrolateral to the AMMC. Smooth dendrites entered the AMMCs of both hemispheres (Figure 10c). In each hemisphere, an axon projected from the dendritic fields into the AL through the antenno‐subesophageal tract (AST). All contralateral glomeruli were densely innervated, while only a few OGs were innervated in the ipsilateral AL. We termed this CN type, Cast.

#### 3.3.2 Unilateral centrifugal neurons

The unilateral CNs consisted of four types: a novel medial‐tract CN called Cm, two novel lateral‐tract CN types, Cl_a and Cl_b, and one CN type resembling a previously reported multisensory CN (Zhao et al., 2013). The last‐mentioned type was termed Csfs, as its protocerebral neurites appeared to follow what Ito et al. (2014) calls the superior fiber system (SFS).

The Cm type was stained in three preparations (Figure 11a,b), one of which was female (CN8). The somata of these neurons were in the posterior cell body rind, dorsoposterior to the PLP. Protocerebral innervations included densely distributed smooth processes in the SCL, LH, VLP, PLP, SLP, SIP, SMP, and the calyces. Additionally, the β‐lobe and the medial branch of the γ‐lobe were sparsely innervated, along with the surrounding crepine. The main neurite branched off near the peduncle and projected anteriorly to the AL along the mALT. Inside the AL, it arborized in all glomeruli. Two of the neurons (CN7 and 8) extended blebby terminals in a cluster of anterior OGs, while the processes in other glomeruli were much smaller and potentially dendritic. The final Cm neuron (CN6) had homogeneous glomerular innervations, where all terminals were varicose. In addition to the main axon, all Cm neurons had a thin branch that followed the lALT from the protocerebrum towards the AL isthmus.

Two CN types, Cl_a and Cl_b, projected from the protocerebrum into the AL through the lALT. The first lateral‐tract CN type, Cl_a, included two labeled neurons, one in a male (CN9; Figure 12) and the other in a female (CN10; Figure 11c). Their somata were in the posterior cell body rind, and both CNs arborized in the VLP and LH. In addition, CN9 had processes in the ICL and SLP, while CN10 innervated the PLP, SIP and the base of the calyces. Both CNs had oligoglomerular innervations in the AL. CN9 targeted VPGs, including G64‐G69, G71‐G72, and the LPOG, while CN10 innervated primarily PCx glomeruli and dorsoposterior OGs. Furthermore, the CNs innervated the AMMC and the SEZ. Here, the projection ended up close to the boundary of the SEZ and the ventral nerve cord. Separating between pre‐ and post‐synaptic sites was complicated as the synaptic sites appeared quite similar across neuropils.

The other lateral‐tract CN type, Cl_b (Figure 11d), included one CN having its soma in the anterior cell body rind, anterior to the VLP. From here, a neurite extended posteriorly and bifurcated into numerous smooth, dendrite‐like branches innervating the VLP, PLP, LH, and SLP. The main axon then projected along the lALT and bifurcated at the AL border. A thin branch innervated medial and ventral OGs with fine processes, whereas the other, thicker branch projected dorsally in the AL. Here, particularly swelled terminals targeted the AL hub, along with peripheral parts of dorsal glomeruli (including MGC, PCx, and OGs) and, primarily, the extraglomerular spaces surrounding these glomeruli.

Finally, we labeled two Csfs neurons (Figure 11e), which had their somata in the cell body rind along the midline (MCBR), medioanterior to the SMP. From here, one of the Csfs neurons (CN12) had a primary neurite projecting to the contralateral hemisphere through the superior AL commissure. This fiber forked once it crossed the midline, sending one neurite to the AL and numerous thick neurites through the crepine, into the SCL, SMP, SIP, and SLP, where they further ramified into many thin and smooth dendrite‐like processes. In the AL, innervations included dense, blebby terminals in one posteriorly positioned OG, and bifurcated processes that ran to the AL hub. The other Csfs neuron (CN13) was fully confined to one hemisphere. Although the entire extent of its multiglomerular AL innervations could not be characterized due to co‐labelling of other AL neurons, the CN appeared identical to the CNs reported by Zhao et al. (2013). They performed mass staining from both ALs and found totally two or three AL‐innervating MCBR somata on either side of the midline. As we identified one contralaterally projecting Csfs neuron, we hypothesized that each hemisphere contains at least one ipsilateral and one contralateral Csfs neuron. Consequently, we performed mass staining experiments from one AL by applying Alexa Fluor 488. In total, three MCBR somata close to the brain's midline were labeled, one ipsi‐ and two contralateral (Figure 11f). Assuming that the only MCBR neurons contacting the ALs are the Csfs neurons, there are likely six such CNs, of which four are contralaterally projecting.

## 4 DISCUSSION

In this work, we present a comprehensive collection of individual AL neurons from the moth brain—including all three categories. Except for a previous report from Tanaka et al. (2012), who mapped AL‐neurons in the fruit fly using genetically modified strains, no corresponding overview of such neurons has been described in any insect species. We found that the 190 stained neurons consisted of 25 distinct neuronal types or sub‐types, many of them novel. Generally, all AL neuron categories consist of various heteromorphic types, suggesting complex olfactory processing already at the first synaptic level of the sensory pathway.

### 4.1 PNs and the organization of parallel antennal‐lobe tracts

The PNs of the moth traverse six distinct ALTs (Homberg et al., 1988; Ian, Zhao, et al., 2016; Namiki & Kanzaki, 2011), all represented in our results. In comparison with mammals, the number of parallel tracts is substantially higher in insects. One reason for this discrepancy may be the fact that insects possess a high proportion of multiglomerular PNs, whereas mammals have only uniglomerular PNs (Buck & Bargmann, 2013).

In moths, most uniglomerular PNs, assumed to be essential to odor discrimination, are confined to the mALT (Hansson et al., 1994; Homberg et al., 1988; Ian, Zhao, et al., 2016; Kanzaki et al., 1989). In this study, about half of all labeled PNs were morphologically homogenous uniglomerular Pm_a neurons, which might be compared with mammalian mitral and tufted cells. Both mammalian and insect uniglomerular PNs innervate higher centers involved in identification of odor quality, that is the piriform cortex (Gottfried, 2010) and calyces (Galizia, 2014), respectively. Our data demonstrate that tracts other than the mALT are formed mainly by axons of multiglomerular PNs, with the exception of the dmALT consisting of bilaterally uniglomerular PNs (Figure 6a). Generally, the multiglomerular PNs do not target the calyces. One central difference between the roles of uni‐ and multiglomerular PNs may relate to combination selectivity. We found that the glomerular innervations of the latter PNs were often distant from the putative spike initiating proximal parts of the dendritic tree (Christensen, D'Alessandro, Lega, & Hildebrand, 2001; Gouwens & Wilson, 2009). As electrophysiological signals attenuate while traveling downstream towards a spike initiation zone (Rall & Rinzel, 1973), these PNs should be most responsive to specific stimuli that activate several of the contacted glomeruli. This is similar to the combination selectivity of pyramidal cells in the mammalian piriform cortex (Kumar et al., 2018), even though it occurs at a lower processing level. While the combination selectivity of a single neuron in mammals is random, it may be hardwired in the multiglomerular PNs of the moth, as indicated by consistent AL innervations of specific PN sub‐types across individual moths.

The output targets vary across PN types, although the LH is innervated by most PNs. In line with former studies, all Pm_a neurons from ordinary glomeruli target the calyces and the LH exclusively (Homberg et al., 1988; Martin et al., 2011). The mostly multiglomerular lALT PNs, on the other hand, project primarily to more ventral neuropils such as the VLP (Figure 3; Homberg et al., 1988; Ian, Zhao, et al., 2016). The Pl_a sub‐type targeting the column of the SIP is not represented here. All stained PNs of this sub‐type innervated the MGC, and they were recently reported in Chu et al. (2020). The almost exclusively multiglomerular mlALT PNs all innervate the SLP (Figure 4), and most also target the LH. The three remaining tracts are, so far, rarely or incompletely described at the level of individual neurons. We found that PNs in the recently identified tALT (Ian, Berg, et al., 2016; Ian, Zhao, et al., 2016; Tanaka et al., 2012) are mostly oligoglomerular and project to the PLP, while other protocerebral targets vary across sub‐types (Figure 5). The labeled dALT PN had widespread axon terminals and connected to large parts of the AL (Figure 6b). As many dALT and mlALT PNs are GABAergic (Berg et al., 2009), they can provide inhibitory input to many protocerebral regions. Finally, the dmALT PNs resemble the mALT PNs in many ways, including innervation of the calyces and LH (Ian, Zhao, et al., 2016; Kanzaki et al., 1989; Rø et al., 2007). However, we found that these uniglomerular PNs only innervated one of the VPGs, had somata located outside the AL, and extended additional terminal branches into the PLP.

#### 4.1.1 Connections between distinct PN types/sub‐types and ventroposterior glomeruli

By investigating the morphological data from many individually stained PNs passing along different tracts, we could describe glomerular arborization patterns across PN types. An interesting observation concerns the VPGs (see definition in section 2.4). These heteromorphic glomeruli were previously found to be substantially weaker labeled by OSN mass dye fills than most other glomeruli (Zhao et al., 2016), indicating that they receive relatively little antennal input. In addition, the LN branching patterns found within the VPGs in this study is distinct from that in the OGs, resembling how the MGC versus OGs arborizations differ. Taken together, this implies that the VPGs may pose its own functional AL sub‐system. In total, 20 of the reported 109 PNs had dendrites completely or primarily confined to the VPGs. Notably, *none* of the 61 labeled Pm_a neurons innervated the VPGs.

Interestingly, all four bilateral dmALT PNs innervated both ALs with tightly packed dendrites in one of two glomeruli, G71 or G72, exclusively (Figure 6a). Up to now, only a few incompletely labeled neurons in this thin fiber bundle, which is estimated to include ca. 16 axons in *M. sexta* (Homberg et al., 1988), has been described. Notably, the two glomeruli are clustered together with the LPOG, which handles input about CO_2_ rather than general odors.

Furthermore, some of the PNs connected with the VPGs appear to combine AL signals with inputs from the AMMC and SEZ. This applies to the oligoglomerular Pm_e neurons (Figure 2c), and the uniglomerular Pt_b neuron (Figure 5b). Other PNs arborizing within the VPGs include additional neuron sub‐types confined to the transverse, lateral, and mediolateral ALTs, that is Pt_a (Figure 5a), Pl_c (Figure 3b), and Pml_b (see PN77 in Figure 7). The fact that these PNs display different connection patterns with the VPGs indicates that the information represented within each specific VPG is subject to distinct encoding schemes across PN sub‐types.

The output areas of the VPG PNs cover various and widespread protocerebral regions. However, they all target the PLP, and most of them also terminate in the adjoining LH. The only PN sub‐type with notable innervation of the PLP without having dendrites in the VPGs were the Pml_c neurons. Comparing our findings with reports from the fruit fly, where seven VPGs process thermo‐ and hygro‐sensory inputs, we find a cross‐species pattern of VPG PNs terminating in the ventroposterior LH and PLP (Frank et al., 2015; Marin et al., 2020). In addition, temperature‐ and humidity‐processing PNs in the cockroach have corresponding morphological traits (Nishino et al., 2003). The relevant glomeruli in these insects are generally heteromorphic, like the moth VPGs, and unlike the spheroidal OGs. PNs innervating corresponding glomeruli in other insects consist of many types, several of which are similar to those reported here. For example, findings from the cockroach include PNs resembling both the Pl_c and Pm_e sub‐types (Figures 7 & 8 in Nishino et al., 2003). In the fruit fly, tALT PNs and an analogue of the dmALT PNs innervate the VPGs. Moreover, some thermo‐ and hygro‐sensory PNs in the fly had dendrites in the gustatory SEZ (Marin et al., 2020), like the moth's Pm_e neurons, indicating integration across sensory modalities.

### 4.2 The ventrolateral protocerebrum—A putative area for multimodal processing

The VLP was innervated by all Pm_e and Pml_d neurons, most lALT sub‐types, and by the unique dALT PN. Altogether, the different PN types and sub‐types projecting to the VLP originate from diverse AL sub‐systems (see Supplemental Table 1), and include PNs innervating the SEZ and AMMC (Pm_e and Pl_d), thereby having the potential to function as multimodal integrators (Homberg et al., 1988; Tanaka et al., 2012). Previously, the VLP of heliothine moths was reported to be innervated by auditory ascending neurons (Pfuhl, Zhao, Ian, Surlykke, & Berg, 2014) and gustatory interneurons (Kvello, Løfaldli, Rybak, Menzel, & Mustaparta, 2009). The VLP may therefore serve as a multimodal integration center. In the fruit fly, the ventral LH and PLP, both localized adjacent to the VLP, are convergence sites for multimodal inputs, including information about aversive odors (Seki et al., 2017), mechanosensation, audition (Dolan et al., 2019), thermo‐ and hygro‐sensation (Frank et al., 2015; Frank et al., 2017; Marin et al., 2020), and gustation (Kim, Kirkhart, & Scott, 2017).

### 4.3 Information processing in CNs

Contrary to the PNs, the CNs modulate AL activity based on input in other brain regions. However, CN processes in the AL are not only presynaptic, they may be postsynaptic as well (Sun, Tolbert, & Hildebrand, 1993; Zhang & Gaudry, 2016), which is also the case for OSNs, LNs, and PNs (Bates et al., 2020; Rybak et al., 2016; Shimizu & Stopfer, 2017; Sun, Tolbert, & Hildebrand, 1997; Tabuchi et al., 2015). This bidirectional processing complicates classification into CN and PN categories. For instance, the Pl_d neuron, PN66 (Figure 3c), appeared to have both pre‐ and post‐synaptic sites in the AL. In this case, soma location determined our classification, as all reported CNs except for the CSDn have somata outside the AL. We report 14 neurons classified as CNs, forming seven distinct CN types. The Cm, Cl_a, and Cl_b are entirely novel types, and no Cast or bilaterally AL‐innervating DAAR neurons have been intracellularly labeled before.

#### 4.3.1 CNs potentially playing a role in olfactory feedback modulation

Several of the CNs identified here, may play a role as olfactory feedback neurons, based on their protocerebral dendrites being localized in odor‐centers. The three labeled DAAR neurons, for example, arborized in the SMP, SIP, crepine, and SLP (Figure 10a). In *B. mori*, a neuron resembling the DAAR neuron, albeit with unilateral AL innervation, was reported to respond to odor stimulation (Namiki, Iwabuchi, Kono, & Kanzaki, 2014). Indeed, olfactory input could be transmitted to the DAAR neuron`s protocerebral branches both via second order PNs (e.g., Pml_d) and via third order olfactory neurons which innervate relevant regions in both moths and fruit flies (Dolan et al., 2019; Namiki et al., 2014). The DAAR neurons labeled here differed from the otherwise comparable DAAR CNs reported in *M. sexta* by innervating a visual neuropil, the AOTU, rather than the LH (Dacks et al., 2012). Three additional CN types, Cl_a, Cl_b, and Cm, all had dendrites in higher olfactory regions, such as the LH and VLP, suggesting that they may act in odor feedback modulation as well.

Interestingly, some of the putative feedback CNs mentioned above may also contribute in local lateral processing within the AL. For instance, the Cl_b neuron (Figure 11d) had fine arborizations in medial OGs, while the dorsal AL and the AL hub received extraglomerular varicose processes, which implies post‐ and presynaptic sites, respectively. As the AL hub contains neurites of most AL neurons (Homberg et al., 1988), extensive synapses here may enable broad neuromodulation directly onto main neurites. Moreover, two of three Cm neurons (Figure 11a‐b) had particularly large varicosities in a cluster of dorsoanterior OGs while innervations in other glomeruli were substantially smaller, indicating modulation of specific odor signals, possibly arising from both local AL processing and top‐down signals from the protocerebrum.

#### 4.3.2 CN morphology indicates memory‐guided AL influence

The Cm neurons had dendrites in the calyces, medial lobes, and various other protocerebral neuropils, and were thus the only CNs with extensive mushroom body innervations (Figure 11a‐b). Given the involvement of these structures in establishment of odor‐memory (reviewed in Heisenberg, 2003; Stopfer, 2014), the Cm neurons might offer memory‐guided information to the AL.

Furthermore, the DAAR and Csfs types both arborized in the crepine, surrounding the medial lobes. The fruit fly's crepine, formerly called anterior inferiormedial protocerebrum (Ito et al., 2014), is innervated by at least 12 types of mushroom body neurons (Tanaka, Tanimoto, & Ito, 2008). Provided that the crepine in moths also connects with the mushroom body, these CN types may also be involved in memory processes.

#### 4.3.3 The Cast neuron may be a mechanosensory peptidergic CN

As demonstrated, the Cast neuron had anatomical features indicative of bilaterally asymmetric AL modulation via antennal‐mechanosensory input (Figure 10c). Mechanosensory responses have been described in AL neurons (Kanzaki et al., 1989; Zhao & Berg, 2010), but mediation of such responses could also arise directly from mechanosensory neurons on the antennae that terminate in the AL (Han, Hansson, & Anton, 2005).

The full morphology of the Cast neuron has not been previously reported, but a similar tachykinin‐immunoreactive (TKir) CN with only contralateral innervations has been presented (Berg et al., 2007). That TKir CN could have been incompletely reconstructed, possibly due to immunolabeling of a mirror‐symmetric CN interfering with neurite‐tracing. One recent report contradicts the presence of a TKir CN, claiming these neurons to bypass the AL on its ventroposterior side (Zhao, Xie, Berg, Schachtner, & Homberg, 2017), again based on immunolabeling. However, our intracellularly labeled Cast neuron does not only fit the relevant morphological description in Berg et al. (2007), but also corresponds to the confocal images of TKir neurons presented in Figure 1B‐D of Zhao et al. (2017).

### 4.4 On local interneuron diversity

The majority of labeled LNs, 60 of 67, was classified as MGC‐AllGs type, innervating most or all glomeruli (see Figures 8 & 9). The fact that 30% of these LNs had substantially sparser innervations in the MGC than in the OGs, is in accordance with findings from *M. sexta* and *B. mori* (Matsumoto & Hildebrand, 1981; Reisenman et al., 2011; Seki & Kanzaki, 2008). This indicates a reduced role for LN‐mediation in the MGC, yet MGC‐PN activity is clearly mediated by GABA (Christensen, Waldrop, & Hildebrand, 1998; Lei, Christensen, & Hildebrand, 2002), which corresponds with the large proportion of GABAergic LNs found across moth species (Berg et al., 2009; Reisenman et al., 2011; Seki & Kanzaki, 2008). The MGC‐AllGs LNs further included 26%, 20%, and 14% with sparse or no innervations in the LPOG, PCx, and VPGs, respectively. Moreover, all labeled oligoglomerular LNs primarily innervated the OGs. As such, OG processing appears to be the principal focus of the LNs.

We also report one novel LN type, that is the MGC‐AllGs‐IST type (Figure 8e), with branches stretching to the AL isthmus. Neurons extending beyond the AL would usually be classified as PNs, however the neurites in question did not appear to be axonal, nor did these LNs sample inputs primarily beyond the AL, like the CNs do. As this LN type had processes in the AL isthmus, it may have dendro‐axonic connections with lALT PNs, which also arborize here (for example, see PN61 in Figure 7; Homberg et al., 1988).

## 5 CONCLUSION

Here, we present a comprehensive collection of AL neurons in the moth *H. armigera*. The extraordinary morphological diversity of these neurons indicates the advanced chemosensory processing taking place already at the initial synaptic level of the olfactory pathway. In addition to the scientific value of documenting the peculiarities and complexity within a small insect brain, the anatomical data may serve as a basis for future studies aiming at exploring physiological characteristics of the various neuron types.

## CONFLICT OF INTEREST

The authors declare no conflict of interest.

## AUTHOR CONTRIBUTIONS

Jonas Hansen Kymre: Conceptualization, data acquisition, analysis, making figures, writing original manuscript, writing final manuscript. Christoffer Nerland Berge: Data acquisition, analysis, revising original manuscript. Xi Chu: Data acquisition, revising original manuscript. Elena Ian: Neuron reconstruction, uploading neurons to the insect brain database, revising original manuscript. Bente G. Berg: Conceptualization, writing original manuscript, writing final manuscript.

### PEER REVIEW

The peer review history for this article is available at https://publons.com/publon/10.1002/cne.25034.

## Supporting information


**SUPPLEMENTARY TABLE 1** Overview of individual projection neurons

**SUPPLEMENTARY TABLE 2** Overview of individual antennal‐lobe local interneurons

## Data Availability

Reconstructed models, along with confocal image stacks, of the reported neuronal types and sub‐types are freely available through the insect brain database (insectbrainDB, 2020). In this article, confocal images of all included neurons are presented, while tables with detailed descriptions of individual neurons are available in the supplementary materials.
