# Supplementary material for: Antennal‐lobe neurons in the moth Helicoverpa armigera: Morphological features of projection neurons, local interneurons, and centrifugal neurons
Source: J Comp Neurol. 2020 Oct 5;529(7):1516–40. doi: 10.1002/cne.25034 (PMC8048870; doi:10.1002/cne.25034)
Supplement: Supplementary file 1 — SUPPLEMENTARY TABLE 1 Overview of individual projection neurons [file CNE-529-1516-s001.pdf]

SUPPLEMENTARY TABLE 1 Overview of individual projection neurons

Type	ID	*N*	Soma	AL innervations	Non-AL innervations	Figure
**mALT**						
Pm_a	PN1	1	LC	UG (VA OG)	Ca; LH	7
Pm_a	PN2	1	MC	UG (A OG); ALH	Ca; LH	7
Pm_a	PN3	1	LC	UG (PCx)	Ca; LH	7
Pm_a	PN4	1	MC	BG (A OG; PCx)	Ca; LH	7
Pm_a	PN5	1	AC	UG (P OG)	Ca; LH	2a
Pm_a	PN6	1	MC	UG (DA OG)	Ca; LH	7
Pm_a	PN7	1	LC	UG (P OG)	Ca; LH	7
Pm_a	PN8	1	MC	UG (PCx)	Ca; LH	7
Pm_a	PN9	1	AC	UG (P OG)	Ca; LH	7
Pm_a	PN10	1	MC	UG (PM OG)	Ca; LH	7
Pm_a	PN11	1	MC	UG (A OG)	Ca; LH	7
Pm_a	PN12	2	LC	UG (A OG)	Ca; LH	7
Pm_a	PN13	1	MC	BG (D OGs)	Ca; LH	2b
Pm_a	PN14	3	MC	UG (DA OG)	Ca; LH	7
Pm_a	PN15	2	AC	OlG (~6 A OGs)	Ca; LH	7
Pm_a	PN16	1	AC	UG (VA OG)	Ca; LH	7
Pm_a	PN17	1	LC	UG (P OG)	Ca; LH	7
Pm_a	PN18	1	LC	UG (VA OG)	Ca; LH	7
Pm_a	PN19	1	LC	UG (VA OG)	Ca; LH	7
Pm_a	PN20	1	AC	UG (V OG)	Ca; LH	7
Pm_a	PN21	1	AC	UG (V OG)	Ca; LH	7
Pm_a	PN22	1	MC	UG (A OG)	Ca; LH	7
Pm_a	PN23	1	LC	UG (P OG)	Ca; LH	7
Pm_a	PN24	2	MC	UG (P OGs)	Ca; LH	7
Pm_a	PN25	1	MC	UG (PCx)	Ca; LH	7
Pm_a	PN26	1	LC	UG (DA OG)	Ca; LH	7
Pm_a	PN27	1	MC	UG (PCx)	Ca; LH	7
Pm_a	PN28	2	MC	UG (P OG)	Ca; LH	7
Pm_a	PN29	1	AC	UG (P OG)	Ca; LH	7
Pm_a	PN30	1	MC	UG (PCx)	Ca; LH	7
Pm_a	PN31	1	MC	UG (A OG)	Ca; LH	7
Pm_a	PN32	1	MC	UG (PCx)	Ca; LH	7
Pm_a	PN33	1	LC	BG (VA OGs)	Ca; LH	7
Pm_a	PN34	1	AC	UG (P OG)	Ca; LH	7
Pm_a	PN35	1	MC	UG (A OG)	Ca; LH	7
Pm_a	PN36	1	AC	UG (P OG)	Ca; LH	7
Pm_a	PN37	1	MC	UG (A OG)	Ca; LH	7
Pm_a	PN38	1	MC	UG (PCx)	Ca; LH	7
Pm_a	PN39	1	MC	UG (PCx)	Ca; LH	7
Pm_a	PN40	1	LC	UG (A OG)	Ca; LH	7
Pm_a	PN41	1	MC	UG (P OG)	Ca; LH	7
Pm_a	PN42	1	AC	UG (P OG)	Ca; LH	7

Pm_a	PN43	2	LC	UG (DA OG)	Ca; LH	7
Pm_a	PN44	1	MC	UG (A OG)	Ca; LH	7
Pm_a	PN45	1	LC	UG (A OG)	Ca; LH	7
Pm_a	PN46	1	LC	BG (A OGs)	Ca; LH	7
Pm_a	PN47	1	LC	UG (A OG)	Ca; LH	7
Pm_a	PN48	1	MC	UG (PCx)	Ca; LH	7
Pm_a	PN49	2	LC	UG (A OGs)	Ca; LH	7
Pm_a	PN50	1	LC	UG (A OG)	Ca; LH	7
Pm_a	PN51	3	LC	UG (A OGs)	Ca; LH	7
Pm_e	PN52	1	AC	OlG (VPGs)	SEZ; AMMC; ICL; SLP; PLP; LH; VLP	7
Pm_e	PN53	1	AC	OlG (VPGs)	ICL; SLP; PLP; LH; VLP	7
Pm_e	PN54	1	AC	OlG (VPGs)	SEZ; AMMC; ICL; SLP; PLP; LH; VLP	7
Pm_e	PN55	1	†	OlG (VPGs; M OGs)	SEZ; AMMC; ICL; SLP; PLP; LH; VLP	7
Pm_e	PN56	1	AC	OlG (VPGs; M OGs)	SLP; PLP; LH †	7
Pm_e	PN57	1	AC	OlG (VPGs; M & A OGs)	SEZ; AMMC; SLP; ICL; PLP; LH; VLP	2c
**lALT**						
Pl_b	PN58	1	LC	UG (VM OG)	VLP	7
Pl_b	PN59	1	LC	OlG (OGs) †	VMNP; VLP	3a
Pl_c	PN60	1	LC	OlG (VPGs; VA, M OGs)	LH; PLP; Ca	7
Pl_c	PN61	1	LC	OlG (VPGs G64-69)	AL Isthmus; LH; PLP; Ca	7
Pl_c	PN62	1	LC	OlG (VPGs G64-69)	LH; PLP; Ca	7
Pl_c	PN63	1	LC	OlG (VPGs G64-69)	LH; PLP; Ca	7
Pl_c	PN64	1	LC	UG (DM OG)	LH; PLP; Ca	7
Pl_c	PN65	1	LC	OlG (VPGs G64-69)	LH; PLP; Ca	3b
Pl_d	PN66	1	LC	OlG (~10 PCx; 5 M OGs)	SEZ; AMMC; VMNP; VLP	3c
Pl_d	PN67	1	LC	OlG (~10 D, V, M OGs)	SEZ; AMMC VMNP; VLP	3d
Pl_d	PN68	1	LC	OlG (~40-50 OGs; PCx)	AMMC; VLP †	7
Pl_d	PN69	1	LC	MGC-AllGs	VMNP; VLP	3e
Pl_d	PN70	1	LC	MGC-AllGs	SEZ; LAL; VMNP; VLP	7
Pl_d	PN71	1	LC	MGC-AllGs	SEZ; LAL; VMNP; VLP	7
Pl_e	PN72	2	LC	UG (PCx)	VLP; LH; SCL	7
Pl_e	PN73	1	LC	OlG (VM OGs; VPGs)	AL Isthmus; VLP; LH	7
Pl_e	PN74	1	LC	OlG (OGs; VPGs)	SEZ; AMMC; VLP; LH; ICL	3f
**mlALT**						
Pml_b	PN75	1	LC	OlG (~4 VPGs)	LH; PLP; SLP; SMP; bCa	7
Pml_b	PN76	1	LC	OlG (~4 PCx; 1 M OG)	LH; PLP; SLP; bCa	4a
Pml_b	PN77	1	LC	OlG (~4 VPGs)	LH; PLP; SLP; SMP; bCa	7
Pml_b	PN78	1	LC	OlG (~4 PCx)	SLP; LH †	7
Pml_c	PN79	1	LC	OlG (10-15 A OGs)	LH; PLP; SLP; ICL; bCa	7
Pml_c	PN80	1	LC	OlG (10-15 A OGs)	LH; PLP; SLP; ICL; bCa	4b
Pml_c	PN81	1	LC	OlG (10-15 A OGs)	LH; PLP; SLP; ICL; bCa	7
Pml_c	PN82	1	LC	†	LH; PLP; SLP; ICL; bCa	7
Pml_c	PN83	1	LC	UG (PCx G49)	LH; PLP; SLP; ICL; bCa	4c
Pml_d	PN84	1	LC	MGC-AllGs	VLP; LH; SLP; SIP; SMP	7
Pml_d	PN85	1	LC	MGC-AllGs	VLP; LH; SLP; SIP; SMP; CRE	4d
Pml_d	PN86	1	LC	MGC-AllGs	VLP; LH; SLP; SIP; SMP; CRE	7
Pml_d	PN87	1	LC	MGC-AllGs	VLP; SLP; SIP; SMP; CRE	7

**tALT**					
Pt_a	PN88	1 LC	OlG (~7 VPGs; G36)	LH; PLP; Ca	5a
Pt_a	PN89	1 LC	OlG (~9 VPGs; G36)	LH; PLP; Ca	7
Pt_a	PN90	1 LC	OlG (~6 VPGs; G36)	LH; PLP; Ca	7
Pt_b	PN91	1 LC	UG (VPG G73)	SEZ; AMMC; BL. Ca, PLP, SLP †	5b
Pt_c	PN92	1 LC	OlG (3 PCx; VPGs)	SLP; PLP	5c
Pt_d	PN93	1 LC	OlG (VPGs; M, D OGs)	SLP; PLP	5d
**dmALT**					
Pdm	PN94	1 †	BL. UG (VPG G72)	Ca; LH; PLP	7
Pdm	PN95	1 GCBR	BL. UG (VPG G72)	SEZ; BL. Ca, LH, PLP	6a
Pdm	PN96	1 †	BL. UG (VPG G71)	Ca; LH; PLP	7
Pdm	PN97	1 GCBR	BL. UG (VPG G71)	Ca; LH; PLP	7
**dALT**					
Pd	PN98	1 MC	CL. MG †	CL. VLP; PLP; LH; bCa; SLP; SIP; SMP	6b

Note: † indicates that full characterization was not possible, either because of co-stained neurons, obscuring elements or weak labelling of certain neuronal compartments. A, anterior; AC, anterior cell cluster; AL; antennal lobe; ALH; antennal-lobe hub; ALT, antennal-lobe tract; AMMC, antennal mechanosensory and motor centre; bCa, base of the calyces; BG, biglomerular; BL., bilateral; Ca, calyces of the mushroom bodies; CL., contralateral; CRE, crepine; D, dorsal; DA, dorsoanterior; dALT, dorsal ALT; dmALT, dorsomedial ALT; Gs, glomeruli; GCBR, cell body rind around the gnathal ganglia; ICL, inferior clamp; LAL, lateral accessory lobe; lALT, lateral ALT; LC, lateral cell cluster; LH, lateral horn; M, medial; mALT, medial ALT; MC, medial cell cluster; MG, multiglomerular; MGC-AllGs, macroglomerular complex and all/most other glomeruli; mlALT, mediolateral ALT; OGs, ordinary glomeruli; OlG, oligoglomerular; P, posterior; PCx, posterior complex glomeruli; PLP, posterior lateral protocerebrum; PM, posteromedial; PN, projection neuron; SCL, superior clamp; SEZ, subesophageal zone; SIP, superior intermediate protocerebrum; SLP, superior lateral protocerebrum; SMP, superior medial protocerebrum; tALT, transverse ALT; UG, uniglomerular; V, ventral; VA, ventroanterior; VLP, ventrolateral protocerebrum; VMNP, ventro-medial neuropil; VPGs; ventroposterior glomeruli.
