# Supplementary material for: Antennal‐lobe neurons in the moth Helicoverpa armigera: Morphological features of projection neurons, local interneurons, and centrifugal neurons
Source: J Comp Neurol. 2020 Oct 5;529(7):1516–40. doi: 10.1002/cne.25034 (PMC8048870; doi:10.1002/cne.25034)
Supplement: Supplementary file 2 — SUPPLEMENTARY TABLE 2 Overview of individual antennal‐lobe local interneurons [file CNE-529-1516-s002.pdf]

SUPPLEMENTARY TABLE 2 Overview of individual antennal-lobe local interneuro[illegible]

Type	ID	*N*	PCx	MGC	LPOG	VPGs	OGs	Figure
**MGC-AllGs**								
	LN1	1	○	○	●	●	●	9
	LN2	1	●	●	●	○	●	9
	LN3	2	●	○	×	○	●	9
	LN4	1	○	●	●	●	●	9
	LN5	2	●	●	○	●	●	9
	LN6	1	●	●	●	●	●	9
	LN7	2	●	●	○	○	●	8c
	LN8	1	●	●	●	●	●	9
	LN9	2	●	●	●	+	●	9
	LN10	1	●	●	●	●	●	9
	LN11	1	○	○	●	●	●	9
	LN12	1	●	●	●	●	●	9
	LN13	2	○	○	●	●	●	9
	LN14	2	●	●	●	●	●	9
	LN15	1	●	●	●	●	●	9
	LN16	1	●	○	●	●	●	9
	LN17	1	●	○	●	●	●	9
	LN18	1	+	●	●	●	●	9
	LN19	1	●	○	●	+	●	9
	LN20	1	●	●	×	●	●	9
	LN21	1	×	○	○	●	●	8b
	LN22	1	●	○	×	○	●	9
	LN23	1	●	●	○	●	●	9
	LN24	1	●	○	●	●	●	9
	LN25	2	●	●	●	●	●	9
	LN26	1	●	○	●	●	●	9
	LN27	1	○	○	●	●	●	9
	LN28	2	●	●	●	●	●	9
	LN29	1	●	●	●	●	●	9
	LN30	1	●	●	—	—	●	9
	LN31	1	●	●	●	●	●	9
	LN32	1	●	●	—	—	●	9
	LN33	1	●	●	●	●	●	9
	LN34	1	○	○	●	●	●	9
	LN35	1	●	●	×	○	●	9
	LN36	1	●	●	—	—	●	9
	LN37	1	●	○	●	●	●	9
	LN38	2	●	●	●	●	●	9
	LN39	1	●	●	●	●	●	9
	LN40	1	●	—	●	●	●	9
	LN41	1	●	●	●	●	●	9
	LN42	1	●	●	○	●	●	8a

	LN43	1	●	●	●	●	●	9
	LN44	1	●	●	○	○	●	9
	LN45	1	●	●	●	●	●	9
	LN46	2	○	○	●	●	●	9
	LN47	2	○	●	○	●	●	9
	LN48	2	●	●	●	●	●	9
**OligoGs**								
	LN49	1	×	×	●	●	●	9
	LN50	1	○	×	×	○	●	9
	LN51	1	×	○	×	×	●	8d
**MGC-AllGs-IST**								
	LN52	1	●	●	●	●	●	8e
	LN53	1	●	●	●	●	●	9
	LN54	2	●	●	○	○	●	9

Note: —, non-discernible; ×, not innervated; ○, sparse innervation; ●, normal innervation; +, dense innervation; IST, antennal-lobe isthmus; LPOG, labial-palp pit organ glomerulus; MGC, macroglomerular complex; MGC-AllGs, macroglomerular complex and all/most other glomeruli; OGs, ordinary glomeruli; OligoGs, oligoglomerular; PCx, posterior complex; VPGs, ventroposterior glomeruli.
